# Copy number amplification-induced overexpression of lncRNA LOC101927668 facilitates colorectal cancer progression by recruiting hnRNPD to disrupt RBM47/p53/p21 signaling

**DOI:** 10.1186/s13046-024-03193-7

**Published:** 2024-09-30

**Authors:** Zaozao Wang, Haibo Han, Chenghai Zhang, Chenxin Wu, Jiabo Di, Pu Xing, Xiaowen Qiao, Kai Weng, Hao Hao, Xinying Yang, Yifan Hou, Beihai Jiang, Xiangqian Su

**Affiliations:** 1 Key laboratory of Carcinogenesis and Translational Research (Ministry of Education/Beijing), Department of Gastrointestinal Surgery IV, Peking University Cancer Hospital & Institute, No.52 Fucheng Road, Haidian District, 100142 Beijing, China; 2 Key laboratory of Carcinogenesis and Translational Research (Ministry of Education/Beijing), Department of Clinical Laboratory, Peking University Cancer Hospital and Institute, No.52 Fucheng Road, Haidian District, 100142 Beijing, China; 3 State Key Laboratory of Holistic Integrative Management of Gastrointestinal Cancers, Beijing Key Laboratory of Carcinogenesis and Translational Research, Department of Gastrointestinal Surgery IV, Peking University Cancer Hospital & Institute, No.52 Fucheng Road, Haidian District, 100142 Beijing, China

**Keywords:** LOC101927668, Colorectal cancer, RBM47/p53/p21 signaling, HnRNPD

## Abstract

**Background:**

Somatic copy number alterations (SCNAs) are pivotal in cancer progression and patient prognosis. Dysregulated long non-coding RNAs (lncRNAs), modulated by SCNAs, significantly impact tumorigenesis, including colorectal cancer (CRC). Nonetheless, the functional significance of lncRNAs induced by SCNAs in CRC remains largely unexplored.

**Methods:**

The dysregulated lncRNA LOC101927668, induced by copy number amplification, was identified through comprehensive bioinformatic analyses utilizing multidimensional data. Subsequent in situ hybridization was employed to ascertain the subcellular localization of LOC101927668, and gain- and loss-of-function experiments were conducted to elucidate its role in CRC progression. The downstream targets and signaling pathway influenced by LOC101927668 were identified and validated through a comprehensive approach, encompassing RNA sequencing, RT-qPCR, Western blot analysis, dual-luciferase reporter assay, evaluation of mRNA and protein degradation, and rescue experiments. Analysis of AU-rich elements (AREs) within the mRNA 3’ untranslated region (UTR) of the downstream target, along with exploration of putative ARE-binding proteins, was conducted. RNA pull-down, mass spectrometry, RNA immunoprecipitation, and dual-luciferase reporter assays were employed to elucidate potential interacting proteins of LOC101927668 and further delineate the regulatory mechanism between LOC101927668 and its downstream target. Moreover, subcutaneous xenograft and orthotopic liver xenograft tumor models were utilized to evaluate the in vivo impact of LOC101927668 on CRC cells and investigate its correlation with downstream targets.

**Results:**

Significantly overexpressed LOC101927668, driven by chr7p22.3-p14.3 amplification, was markedly correlated with unfavorable clinical outcomes in our CRC patient cohort, as well as in TCGA and GEO datasets. Moreover, we demonstrated that enforced expression of LOC101927668 significantly enhanced cell proliferation, migration, and invasion, while its depletion impeded these processes in a p53-dependent manner. Mechanistically, nucleus-localized LOC101927668 recruited hnRNPD and translocated to the cytoplasm, accelerating the destabilization of RBM47 mRNA, a transcription factor of p53. As a nucleocytoplasmic shuttling protein, hnRNPD mediated RBM47 destabilization by binding to the ARE motif within RBM47 3'UTR, thereby suppressing the p53 signaling pathway and facilitating CRC progression.

**Conclusions:**

The overexpression of LOC101927668, driven by SCNAs, facilitates CRC proliferation and metastasis by recruiting hnRNPD, thus perturbing the RBM47/p53/p21 signaling pathway. These findings underscore the pivotal roles of LOC101927668 and highlight its therapeutic potential in anti-CRC interventions.

**Supplementary Information:**

The online version contains supplementary material available at 10.1186/s13046-024-03193-7.

## Introduction

Colorectal cancer (CRC) ranks as the third leading cause of cancer-related mortality worldwide [1]. Despite substantial progress in treatment modalities such as surgery, adjuvant or neoadjuvant chemotherapy, targeted therapy, and immunotherapy in recent decades, patients diagnosed with advanced stages of CRC still face unfavorable prognoses due to diagnostic limitations, early metastasis or recurrence, and drug resistance [2]. Hence, there is a critical need to elucidate the molecular mechanisms underlying CRC progression and to identify novel biomarkers that can facilitate early detection, enhance therapeutic efficacy, and ultimately improve patient prognosis.

RNA-binding proteins (RBPs) are pivotal regulators of post-transcriptional gene expression, with approximately 1542 RBPs encoded by the human genome [3–5]. Among these, RBM47 is a conserved RBP in vertebrates and exerts essential roles in gene regulation [6]. Functioning as a multifaceted RBP, RBM47 is involved in diverse biological and pathological processes, including early embryonic development, C to U RNA editing, and tumor suppression [7]. Decreased expression of RBM47 has been implicated in promoting proliferation and metastasis across various cancers, including breast cancer, lung adenocarcinoma, and CRC [8–10]. RBM47 acts as a crucial tumor suppressor by activating the p53/p21 signaling pathway through promotion of p53 transcription, and it also negatively regulates the Wnt/β-catenin signaling pathway [8, 11]. Additionally, RBM47 inhibits Nrf2 activity, thereby suppressing lung adenocarcinoma tumor growth [9], and enhances IL-10 production while inhibiting B-cell immunity at the post-transcriptional level [12]. Notably, during epithelial-mesenchymal transition (EMT), the transcription of RBM47 is suppressed by IL6-activated STAT3 or ectopic overexpression of SNAIL and SLUG, through binding to conserved binding sites within the RBM47 promoter region [10]. However, the precise mechanisms underlying RBM47 downregulation in tumors remain largely elusive.

Long non-coding RNAs (lncRNAs), characterized as transcripts exceeding 200 nucleotides that lack protein-coding capacity, exert crucial regulatory functions in diverse pathophysiological processes by modulating gene expression at epigenetic, transcriptional, or post-transcriptional levels [13, 14]. Their remarkable stability in physiological fluids such as blood and urine, coupled with resistance to nuclease-mediated degradation, renders lncRNA expression levels promising as reliable indicators of disease severity [15]. Given the significant potential of lncRNAs in both tumor diagnosis and treatment, identifying key lncRNAs involved in tumor initiation and progression, along with elucidating their underlying molecular mechanisms, is crucial for uncovering their clinical value in translational therapy.

Cancer genomes frequently harbor somatic copy number alterations (SCNAs), which can lead to the overexpression of oncogenes or the inactivation of tumor suppressor genes, thereby exerting a profound impact on cellular processes [16]. Approximately 21.8% of lncRNA genes are situated in regions characterized by focal SCNAs [17]. Recent studies have elucidated the critical roles of deregulated lncRNAs induced by SCNAs in carcinogenesis [18]. For instance, increased expression of FAL1 due to copy number amplification of chromosome 1, represses p21 transcription by binding to the epigenetic repressor BMI1 [17]. Furthermore, dysregulated lncRNAs resulting from copy number variations (CNVs) have been associated with unfavorable prognosis in cancer patients, including prostate cancer and CRC [19, 20].

Our previous study established CNV-lncRNA-mRNA regulatory triplets to delineate CNV-based gene dosage effects and identify key fragile sites in cancer genomes using a multidimensional dataset [21]. Considering that 53% of genes affected by CNVs were influenced by distal CNVs not directly adjacent to the target genes [22], we reanalyzed the expression differences and correlations between lncRNA and mRNA transcripts along with CNVs. The goal was to identify dysregulated lncRNAs located within SCNAs that are essential for CRC progression. It was observed that a worse prognosis was associated with LOC101927668, an aberrantly overexpressed lncRNA caused by frequent copy number gain of chr7p22.3-p14.3 in CRC patients. This lncRNA facilitated the proliferation and metastasis of CRC cells by suppressing RBM47/p53/p21 signaling. Mechanistically, the overexpression of LOC101927668 accelerated the degradation of RBM47 mRNA through its interaction with heterogeneous nuclear ribonucleoprotein D (hnRNPD), an AU-rich element (ARE)-binding protein known for its crucial roles in mRNA stability regulation. In conclusion, this study demonstrates that the lncRNA LOC101927668, located within SCNAs, disrupts RBM47/p53/p21 signaling through its interaction with hnRNPD, thereby accelerating the progression of CRC.

## Materials and methods

### Human subjects

Ninety-two paired samples of CRC and adjacent normal tissues were collected from patients who underwent surgical resection at the Department of Gastrointestinal Surgery IV, Peking University Cancer Hospital & Institute between 2012 and 2015. None of the patients received preoperative chemotherapy or chemoradiotherapy. The study was conducted following the principles outlined in the Declaration of Helsinki and was approved by the Ethics Committee of Peking University Cancer Hospital & Institute (No. 2020KT127). Written informed consent was obtained from all participating patients.

## Cell culture

Human colonic epithelial and CRC cell lines, including HcoEpic, NCM460, RKO, HCT116, HT29, SW480, and LOVO, as well as human embryonic kidney cell line HEK293T were obtained from the American Type Culture Collection (ATCC, Manassas, VA, USA). The cell lines were cultured in Roswell Park Memorial Institute 1640 medium (RPMI 1640) or Dulbecco’s Modified Eagle Medium (DMEM), supplemented with 10% fetal bovine serum (FBS) and 1% penicillin/streptomycin. Cells were maintained in a humidified atmosphere containing 5% CO_2_ at 37 °C, following established protocol. Regular screening for mycoplasma contamination was performed every six months, and the authenticity of cell lines was confirmed via short tandem repeat (STR) profiling.

## Plasmids, siRNAs, and lentiviral infection

To establish stable cell lines with either overexpression or knockdown of LOC101927668, the full-length transcript of LOC101927668 was inserted into the plenti6 plasmid for overexpression (LOC_OE/puromycin) (Sangon Biotec, Shanghai, China), while short hairpin RNA (shRNA) targeting LOC101927668 (shLOC/puromycin, Table S1) was cloned into the plenti6-U6 vector (NC1/puromycin) for knockdown (GenePharma, Shanghai, China). Lentiviruses were generated from 293 T cells by transfection with packaging plasmids (pLP1, pLP2, pLP-VSVG, Invitrogen). Subsequently, CRC cells of interest were transduced with lentivirus and subjected to puromycin selection to establish stable cell lines with the desired expression levels of LOC101927668. For transient overexpression of full-length LOC101927668 (LOC-FL) and its truncated fragment containing the hnRNPD binding motif (LOC-Δ2, 196–373 bp), both the full-length and truncated fragments of LOC101927668 were synthesized and subcloned into the EcoRI/XhoI sites of the pcDNA3.0 vector (Table S2). The mutant plasmid with point mutations in hnRNPD binding sites (LOC-Δ2-mut) was synthesized and constructed by Sangon Biotech. RBM47 shRNA plasmids (shRBM47/Neomycin), hnRNPD siRNA oligos, together with their corresponding negative controls NC2 (pGPU6/Neomycin) and siNC were obtained from GenePharma (Table S1). Cells were seeded and transiently transfected with the designated plasmids or siRNAs using Lipofectamine 2000 (Invitrogen, USA), following the manufacturer's instructions.

## CCK8, cell cycle, EDU and colony formation assay

Cell viability was assessed using the CCK8 kit (Dojindo, Japan) following the manufacturer's instructions. Absorbance values were measured at 450 nm using a microplate reader (Bio-Rad, Hercules, CA, USA).

Cell cycle of indicated cells was examined by flow cytometry after staining with propidium iodide (PI)/RNase staining buffer (BD Pharmingen, USA).

The effect of LOC101927668 on cell proliferation was assessed using the EdU incorporation assay with the Cell-Light™ EdU In Vitro Imaging kit (RiboBio, Guangzhou, China). Cells were incubated with 50 μM EdU for 2 h at 37 °C, followed by fixation and permeabilization. Proliferative activity was visualized using a Zeiss confocal microscope LSM 700 (Carl Zeiss AG, Germany) after incubation with 1 × Apollo reaction cocktail and staining with Hoechst 33,342. Five fields per well were selected for counting, and each group was performed in triplicate.

For the colony formation assay, cells were seeded at a density of 300–500 cells per well in six-well plates and cultured in complete medium. After two weeks, colonies were fixed with 100% cold methanol and stained with 1% crystal violet. Colonies were then counted after washing with PBS.

## Wound healing and transwell assays

The capacity of the cells to migrate was evaluated using a wound healing test. The target cells were cultivated until they attained over 90% confluence after being put into 6-well plates. Next, a 200-µl plastic pipette tip was used to make a scratch in the cell monolayer. After that, the cells were grown in media without serum. ImageJ software was used to measure the wound widths, and a microscope (Leica Microsystems, Wetzlar, Germany) was used to image the wound gaps at 0 and 48 h after the wound.

To conduct tests on invasion and migration, Boyden chambers with 8-μm pore-sized polycarbonate membranes (Costar, USA) were utilized. Before performing the cell invasion experiment, Boyden chambers were coated with matrigel (5 mg/ml). Serum-free media was used to seed cells into the top chamber, and 700 μL of medium supplemented with 15% FBS was used to fill the bottom chamber. The membranes were fixed and stained with 0.1% crystal violet after incubation. A microscope with five fields of view was used to count the quantity of migrating or invaded cells (Leica Microsystems, Wetzlar, Germany).

## RNA isolation and reverse transcription-quantitative PCR (RT-qPCR)

Total RNA was extracted from freshly cultured cells or tissues, which were ground in liquid nitrogen, using TRIzol reagent (Invitrogen; Thermo Fisher Scientific, Inc.). The RNA samples were then quantified using a NanoDrop spectrophotometer (ND-1000, Thermo Fisher Scientific, Inc.). Subsequently, 1 μg of total RNA was reverse transcribed into cDNA using a Reverse Transcription Kit (Promega Corp.).

The resulting cDNA templates were mixed with corresponding primers (Table S2) and SYBR Green PCR Master Mix (Toyobo Life Science, Osaka, Japan). Quantitative PCR (qPCR) was performed using the ABI 7500 Real-time PCR System (Applied Biosystems; Thermo Fisher Scientific, Inc.), with each reaction conducted in triplicate.

## In situ hybridization (ISH) and fluorescence in situ hybridization (FISH)

To ascertain the subcellular localization of LOC101927668 and RBM47 transcripts (primary and mature mRNA), digoxigenin-labeled antisense riboprobes (BOSTER, Wuhan, China) were employed in conjunction with an In Situ Hybridization Detection Kit (BOSTER, Wuhan, China) for either ISH or FISH analysis. Cells or frozen sections were fixed in 4% paraformaldehyde with 0.1% DEPC, treated with 3% H_2_O_2_, and digested with a proteinase K solution, followed by incubation with a prehybridization solution. Subsequently, samples were hybridized with a reaction buffer containing either LOC101927668, RBM47, U6, or 18S probes overnight at 37 °C in a humidified chamber. Following hybridization, slides were washed using SSC solutions with gradually decreasing concentrations and then blocked with 1% BSA. Biotin-labeled anti-digoxigenin antibody was applied to the slides and washed. For FISH analysis, FITC-labeled anti-Biotin antibody (Jackson ImmunoResearch) was utilized, with cell nuclei counterstained with DAPI for 10 min in the dark. Fluorescence images were captured using a Leica SP5 confocal system. For the ISH assay, the streptavidin–biotin-peroxidase complex (SABC-POD) method was employed instead of using FITC-labeled anti-biotin antibody, followed by chromogenic detection with DAB. After staining, two experienced pathologists employed a semi-quantitative scoring system to determine the ISH staining score. The expression level of LOC101927668 was defined by multiplying the proportion of positively stained cells (≤ 5% = 0; 6–25% = 1; 26–50% = 2; 51–75% = 3; 76–100% = 4) by the staining intensity (no staining = 0; weak staining = 1; moderate staining = 2; strong staining = 3).

## RNA immunoprecipitation (RIP)

To perform RIP, the Magna RIP RNA-Binding Protein Immunoprecipitation Kit (Millipore, USA) was employed according to the manufacturer's instructions. Harvested cells were lysed using RIP lysis buffer containing a protease inhibitor cocktail. The supernatants were then incubated with magnetic beads conjugated with either the hnRNPD antibody (Abcam, ab61193) or IgG (Millipore, AP101) to immunoprecipitate the protein-RNA complex overnight at 4 °C. DNase I and proteinase K, both RNase-free, were consecutively used to eliminate DNA and protein from the RIP complex. The retrieved RNA was purified and reverse transcribed into cDNA, followed by qPCR to detect the enrichment of either LOC101927668 or RBM47.

## In vivo and in vitro RNA pull-down assay

To perform an in vivo RNA pull-down assay, HEK293 cells were co-transfected with LOC101927668-6xMS2bs plasmids, containing six repeated MS2-binding site RNA sequences, and MS2 expression plasmids with Flag tags. After harvesting and lysing the cells, the corresponding complexes with undetermined binding proteins of LOC101927668 or its control were isolated using anti-Flag-conjugated magnetic beads (Sigma). The isolated protein complexes were then separated by SDS-PAGE, and the gel was stained using a PAGE Gel Silver Staining Kit (Solarbio, G7210). Specific bands were excised, analyzed by mass spectrometry, and processed via the Mascot search engine, as previously described [23].

The biotin-labeled sense and antisense strands of LOC101927668 or truncated LOC101927668 were synthesized through in vitro transcription using the MAXIscript™ SP6/T7 Transcription Kit (Invitrogen, AM1320) in conjunction with Pierce™ RNA 3' End Desthiobiotinylation Kit (Thermo Fisher, 20,163). DNA templates for sense, antisense, or truncated LOC101927668 fragments were generated via PCR with gene-specific primers containing T7 or SP6 promoter sequences (Table S2). RNA pull-down assays were conducted using these purified biotinylated transcripts with the Pierce™ Magnetic RNA–protein Pull-down kit (Thermo Fisher, 20,164), following the manufacturer's instructions. The biotin-labeled RNA was initially immobilized on streptavidin magnetic beads and subsequently incubated with the specified protein extracts to form an RNA-binding protein complex. The resulting mixture containing the RNA-binding protein complex was subjected to washing, elution, and heat treatment. The retrieved proteins were then analyzed using Western blot analysis.

## Analysis of mRNA stability

To investigate the degradation of mRNA in the cells, actinomycin D (5 μg/mL) was added to the culture media in order to prevent the synthesis of new RNA. At certain intervals, the treated cells were collected, and RNA was extracted. To measure the expression levels of the target mRNA, RT-qPCR was then performed.

## RNA sequencing

Total RNA was isolated using Trizol reagent (Invitrogen). RNA sequencing was performed by Novogene (Beijing, China) on the Illumina sequencing platform. Quality assessment of the RNA included evaluations for degradation, contamination, purity, and integrity. The NEBNext® Ultra™ RNA Library Preparation Kit was utilized for generating the sequencing libraries. Following cluster formation, an Illumina NovaSeq platform was employed for sequencing the library preparations, resulting in the generation of paired-end reads of 150 bp.

## Dual-luciferase reporter assay

Sequences containing the wild-type RBM47 3'UTR with ARE motifs and its mutated variant with A > C and U > G mutations were synthesized by Sangon Biotech (Shanghai, China) and subsequently incorporated into the 3'UTR region of the pGL3-control vector (Promega). Following this, HCT116 cells were co-transfected with luciferase plasmids and either hnRNPD-targeting siRNAs or their corresponding negative control. Luciferase activity was evaluated using the Dual-Luciferase Reporter Assay System (Promega), with firefly luciferase activity normalized to renilla luciferase activity.

Additionally, to examine the potential regulatory effect of LOC101927668 on the transcriptional expression of RBM47, a fragment of the RBM47 promoter (-2000 bp to 0 bp) was inserted into the pGL4.17 vector (Promega). Dual-luciferase activity was assessed subsequent to co-transfection of HEK293T cells with either the pGL4.17-RBM47 promoter plasmid along with LOC101927668 overexpression or a control vector.

## Cycloheximide (CHX) chase assay

HCT116 cells stably transfected with either LOC101927668 or its corresponding control plasmid were cultured in complete medium supplemented with 100 μg/mL of CHX for the specified duration. Subsequently, cell lysates were subjected to Western blot analysis to assess RBM47 degradation.

## Cytoplasmic and nuclear protein extraction

Proteins from the nuclear and cytoplasmic compartments were separated and extracted using the Nuclear and Cytoplasmic Protein Extraction Kit (Beyotime, P0028, China), with Histone H3 and GAPDH as internal controls respectively.

## Western blot

Protein samples were harvested, separated on SDS–polyacrylamide gels, and then transferred to polyvinylidene difluoride membranes (Millipore). The membranes were subsequently incubated with primary antibodies specific for RBM47 (Abcam, ab167164), p53 (Cell Signaling Technology, 48,818), p21 (Cell Signaling Technology, 2947), hnRNPD (Abcam, ab61193), Histone H3 (Cell Signaling Technology, 4499), GAPDH (Proteintech, 10,494–1-AP) and β-actin (Sigma-Aldrich, A1978), followed by incubation with horseradish peroxidase-conjugated secondary antibodies. Protein bands were visualized using an enhanced chemiluminescence detection reagent (Pierce), and the band intensity was quantified using ImageJ software.

## Immunohistochemistry (IHC)

The expression levels of RBM47 and Ki67 in xenografts and metastatic liver samples were assessed via IHC. Briefly, tumor Sects. (4 μm) were deparaffinized using xylene, gradually rehydrated with decreasing concentrations of ethanol, and treated with 0.3% H_2_O_2_ for 30 min to block endogenous peroxidase activity. After washing and blocking, the sections were incubated overnight at 4 °C with monoclonal antibodies against RBM47 (Abcam, ab167164) or Ki-67 (Abcam, ab16667). Subsequently, the sections were washed and incubated with HRP-conjugated anti-rabbit or anti-mouse IgG antibodies (ZSGB-BIO, China). Immunostaining was visualized using the DAB kit (ZSGB-BIO, China), and nuclear counterstaining was performed using hematoxylin.

## Animal models

All animal experiments conducted in this study received prior approval from the Animal Ethics Committee of Peking University Cancer Hospital and were performed in accordance with the guidelines outlined in the Experimental Animal Management Ordinance, ensuring strict compliance with ethical standards.

To investigate the impact of LOC101927668 on tumor growth in vivo, we injected HCT116 cells that stably overexpressed LOC101927668 or the vector (LOC_OE, Vector), as well as LOVO cells with stable silencing of LOC101927668 (NC1, shLOC_1, shLOC_2), into the hind flanks of 5-week-old female BALB/c-nu/nu mice (Hua-Fu-Kang Corporation, Beijing, China). Moreover, this study also comprehensively assessed the effects of attenuated RBM47 on LOVO cells exhibiting down-regulated LOC101927668 expression in vivo. Tumor growth was monitored every three days using calipers, and tumor volume was calculated using the formula: length × width^2^/2. At the end of 28 days post-tumor inoculation, all mice were euthanized to harvest tumor xenografts, and their weights were recorded.

The effect of RBM47 stably knockdown on LOVO cells with depleted LOC101927668 was also investigated using an orthotopic liver xenograft tumor model. Luciferase-expressing cells (5 × 10^6) were injected into the spleens of mice to establish the model. Metastatic status was evaluated by administering 100 μL of luciferin substrate intraperitoneally for in vivo imaging after anesthesia. Bioluminescence imaging was performed using the IVIS Spectrum In Vivo Imaging System (PerkinElmer, Hopkinton), and average radiance was quantified using Living Image software. Mice were euthanized four weeks post-injection, and metastatic livers were harvested, fixed with 4% PFA, and the number of metastatic nodules was counted. Tissues were then embedded in paraffin for hematoxylin and eosin (HE) and IHC staining. The experiment was conducted in a blinded manner to ensure unbiased results.

## Bioinformatic analysis

The expression profiles of mRNAs and lncRNAs showing significant differential expression in GSE184093 (log_2_ |fold change (FC)|≥ 1, adjusted P-value ≤ 0.05) were visualized through volcano plots. To identify lncRNAs dysregulated by copy number variations (CNVs), we utilized a Venn diagram to identify the intersection between differentially expressed lncRNAs and SCNA foci from patients. The lncRNA-mRNA co-expression network was analyzed using Pearson correlation integrated with Cytoscape software [24]. Data preprocessing, extraction, and visualization (including volcano plots, Venn diagrams, clustered heatmaps, and Circos plots) were performed using relevant R packages including "pheatmap", "clusterProfiler", and the "enrichplot" package [25–27]. Additionally, KEGG pathway analysis was conducted to elucidate potential biological pathways associated with the identified genes.

Expression data for LOC101927668 and RBM47, along with their corresponding clinical information, were retrieved from the Cancer Genome Atlas (TCGA) database and Gene Expression Omnibus (GEO) datasets (GEO9348, GEO21510, GEO41328, and GEO40967) [28–31]. The processing of these datasets utilized several R packages, including "limma", "GEOquery", "TCGAbiolinks", along with "dplyr" and "ggplot2" [32–36]. Additionally, GISTIC2.0 software was employed to identify CNV profiles in 976 CRC samples obtained from TCGA [37].

Copy number data for the CRC cell lines were downloaded from the Cancer Cell Line Encyclopedia (CCLE), which were generated using the Affymetrix SNP 6.0 platform [38]. For each genomic segment, the corresponding chromosome number, base pair range, and mean copy number (segment mean, SM) were obtained from the CCLE data. SM was calculated using the formula SM = log_2_(Copy number/2), focal amplifications were defined as segments with a mean copy number > 0.3, and deletions as those with an SM < -0.3 [39–41]. To clarify the CNV status of CRC cell lines, the raw CCLE data was further processed and visualized using GISTIC 2.0 [37] and the "dplyr", "maftools" and "ggplot2" packages in R [35, 36, 42].

## Statistical analysis

The data were analyzed using SPSS 20.0 or R software. Continuous variables were presented as means with standard deviations. Statistical differences between two groups were assessed using the nonparametric Mann–Whitney U test or the two-tailed unpaired/paired Student t-test. Two-way ANOVA was used to examine cell viability in relation to treatment and time course, followed by one-way ANOVAs with Bonferroni post hoc tests for multiple comparisons. Associations between the expression of LOC101927668 and clinicopathologic features were examined using χ^2^ tests. Pearson correlation analysis was employed to evaluate the correlation between the expression of LOC101927668 and RBM47. Kaplan–Meier analysis and log-rank tests were performed to evaluate the relationship between LOC101927668 expression and patient overall survival. Univariate and multivariate survival analyses were conducted using Cox proportional hazard regression models. A two-tailed *P*-value < 0.05 was considered statistically significant. All experiments were performed in triplicate.

## Results

### LOC101927668, a dysregulated SCNA-harbored lncRNA, was identified in CRC tissues by multi-dimensional data mining

**Fig. 1:**
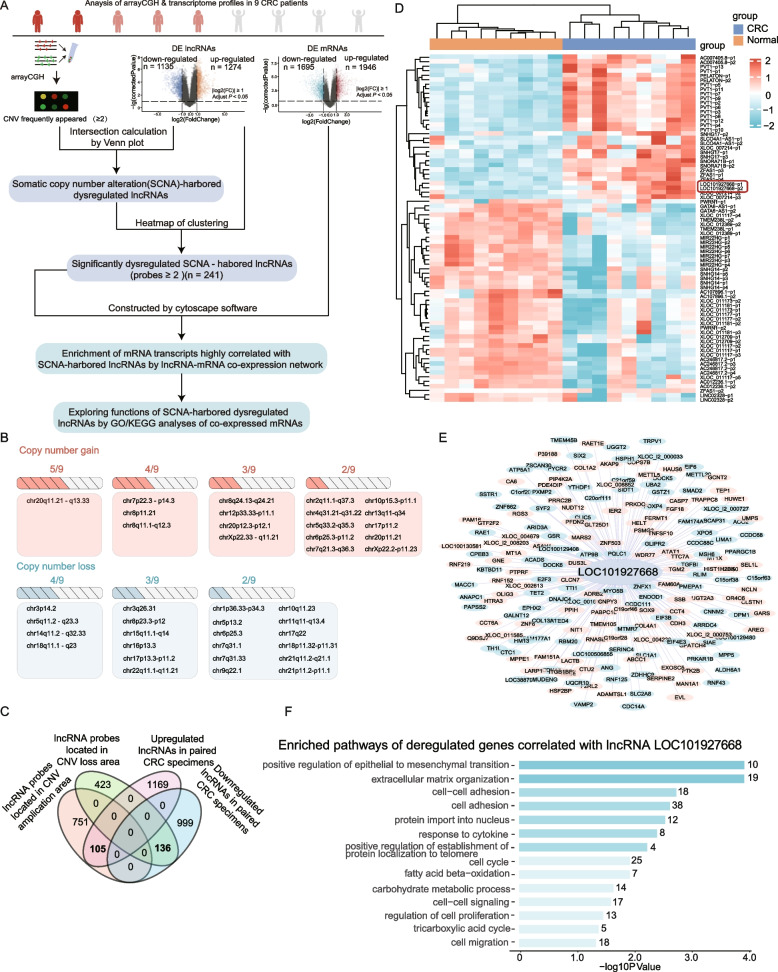
Identification and functional exploration of aberrant SCNA-induced LOC101927668 in CRC through multi-omics integration analysis. **A** Schematic representation of the analytical pipeline for the identification and functional exploration of aberrant SCNA-induced lncRNAs in CRC, utilizing a multidimensional data cohort. DE lncRNAs (mRNAs): differentially expressed lncRNAs (mRNAs). **B** Distribution of focal copy number gain and loss events with high frequency observed in 9 CRC patients (≥ 2/9). **C** Venn diagram illustrating the number of overlapping lncRNAs that were both overexpressed or downregulated and concurrently located within regions of copy number gain or loss. **D** Heatmaps depicting the expression abundance of 24 deregulated SCNA-driven lncRNAs with at least 2 indicative probes, LOC101927668 was highlighted with a red box. **E** Construction of the lncRNA-mRNA co-expression network of LOC101927668 with corresponding mRNA transcripts using Cytoscape. **F** KEGG analysis elucidating the functional annotations of deregulated genes significantly correlated with LOC101927668

**Fig. 2:**
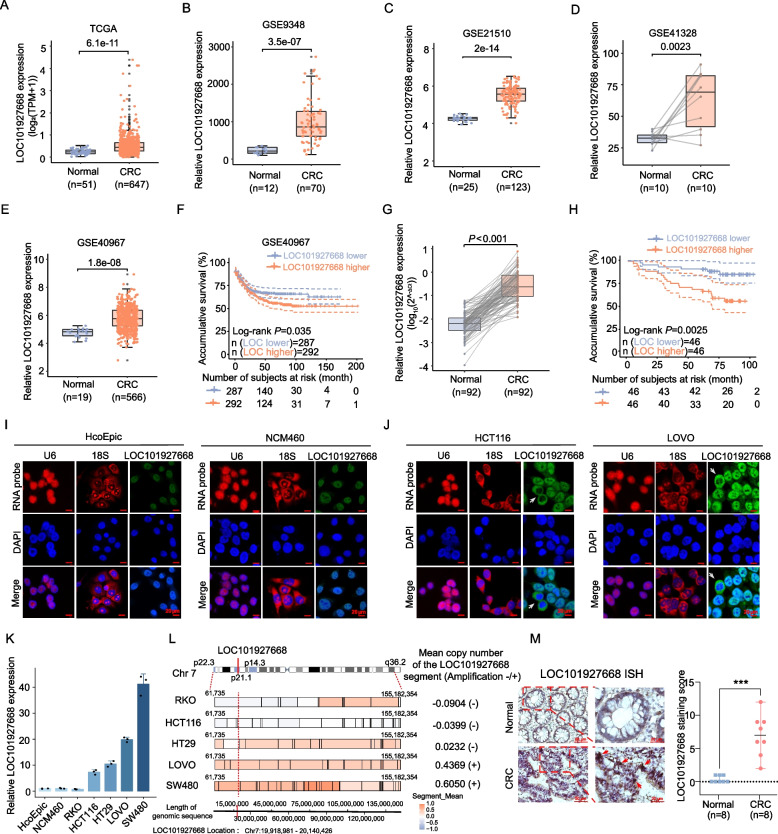
The expression and clinicopathological correlations of LOC101927668 in CRC tissues, along with its subcellular localization and copy number status in CRC cell lines. **A-E** Expression levels of LOC101927668 in normal mucosa and CRC tissues analyzed across different datasets: TCGA (**A**), GSE9348 (**B**), GSE21510 (**C**), GSE41328 (**D**), and GSE40967 (**E**). **F** Kaplan–Meier analysis and log-rank test utilized to assess the relationship between LOC101927668 expression and overall survival of CRC patients in the GSE40967 dataset. **G** RT-qPCR analysis of LOC101927668 expression in 92 paired CRC tissues and adjacent noncancerous tissues. **H** Kaplan–Meier analysis of overall survival with log-rank test in 92 CRC patients stratified by LOC101927668 expression in our own cohort. **I-J** LOC101927668 RNA FISH staining in normal colonic epithelial cell lines HcoEpic and NCM460 (**I**), as well as in CRC cell lines HCT116 and LOVO (**J**), with U6 and 18S serving as positive controls. **K** RT-qPCR analysis of LOC101927668 expression in normal colonic epithelial and CRC cell lines (HcoEpic, NCM460, RKO, HCT116, HT29, LOVO, and SW480). **L** The CNV status of chromosome 7 in CRC cell lines, downloaded from the CCLE, was analyzed and visualized using R. A segment mean > 0.3 was used to indicate copy number amplification, with the location of LOC101927668 highlighted by a red dashed line. **M** Detection of LOC101927668 by ISH staining in 8 paired normal colon epithelia and CRC tissues, with a dot plot showing the distributions of the semi-quantitative ISH scores

Our previous research, based on microarray-based expression chips (GSE184093) and copy number aberration analysis, has elucidated the gene dosage effects of CNV on both mRNA and lncRNAs [21]. To gain comprehensive insights into the complex regulatory networks and biological behaviors of CRC, we extended our analysis to investigate CNV-induced dysregulated lncRNAs, which were pivotal in colorectal cancer progression. The data mining pipeline was depicted in Fig. 1A. Recurrent SCNAs (≥ 2/9) in this cohort (Fig. 1B and Fig. S1A) were consistent with previous reports [43, 44] and those from TCGA-CRC copy number data using GISTIC (Fig. S1B). Dysregulated lncRNAs resulting from genomic aberrations are likely to play crucial roles in tumor development [45]. Therefore, significantly dysregulated lncRNAs associated with frequently appearing CNVs were identified (Fig. 1C and Fig. S1C). Out of 105 upregulated lncRNA probes in copy number gain loci and 136 downregulated lncRNA probes in copy number loss loci, 24 deregulated lncRNAs, each with at least two indicative probes, were further enriched. The expression abundance of each probe for these 24 lncRNAs was displayed in paired normal and CRC specimens (Fig. 1D). Of the nine upregulated lncRNAs located in copy number amplification regions, five were on chr20q11.21-q13.33, two on 8q24.21, and the remaining two on 7p21.1 and 2q31.1 (Table S3). Since the effects and mechanisms of five of these lncRNAs have already been documented in CRC (Table S3), we examined the expression and clinicopathological features of the remaining four unreported upregulated lncRNAs using public databases (Fig. 2A-2F, Fig. S2 and Fig. S3). To explore the potential biological functions of the lncRNA of interest, a lncRNA-mRNA co-expression network was constructed. Differentially expressed mRNAs (|FC|≥ 1.5) that were highly correlated with the lncRNA (R ≥ 0.7, *P*_adjust_ < 0.05) were then enriched and subjected to Gene Ontology (GO) and Kyoto Encyclopedia of Genes and Genomes (KEGG) analyses. Following our analytical protocol, LOC101927668, an upregulated lncRNA due to chr7p22.3-p14.3 amplification, was identified for its significant tumor-promoting potential in CRC (Fig. 1E and F).

## LOC101927668, primarily localized in the nucleus, was upregulated in CRC and associated with poorer prognosis.

Data from TCGA and GEO indicated that LOC101927668 was highly expressed in CRC tissues compared with nontumorous colon epithelia (Fig. 2A-E, Fig. S3A). Furthermore, overexpression of LOC101927668 was closely linked to advanced pathological stage and poorer prognosis (Fig. 2F, Fig. S3B-3D). LOC101927668 expression was further evaluated in a cohort of 92 paired CRC and normal specimens from our department. Elevated LOC101927668 expression in tumors not only indicated poorer overall survival according to Kaplan–Meier analysis (Fig. 2G and H) but also correlated with lymph node and distal metastasis, as well as advanced pathological stage (Table 1). Univariate and multivariate analyses revealed that higher LOC101927668 expression was an independent risk factor for the poorer survival of CRC patients (Table 2). Examination of LOC101927668 in normal colonic epithelial and CRC cell lines by using FISH and RT-qPCR unveiled the upregulated expression of LOC101927668 in CRC cell lines compared to normal counterparts (Fig. 2I-K). In interphase cells, although LOC101927668 was predominantly localized in the nucleus, a small fraction of fluorescent signals was also detected in the cytoplasm (Fig. 2I and J). During mitosis, however, LOC101927668 signals were primarily found in the cytoplasm, such translocation indicating LOC101927668 was dissociated from mitotic chromosomes and returned to the newly formed nuclei in daughter cells (highlighted by gray arrows in Fig. 2J). Furthermore, LOC101927668 expression was significantly higher in CRC cells with chr7p21.1 copy number amplification than those with relatively normal copy number arrangements (Fig. 2K and L, Fig. S4). Consistent with the findings in CRC cell lines, the probes of LOC101927668 were mainly detected in the nuclei of cancerous tissues rather than normal colon epithelia by RNA ISH or FISH assays (Fig. 2M, Fig. S5).

**Table 1:** Correlations between LOC101927668 expression and clinicopathological characteristics in CRC patients

Variables	Cases	LOC101927668 expression		*P*‐value
		Low	High	
*Age (years)*				0.834
≤ 60	50	26	24	
> 60	42	20	22	
*Gender*				0.086
Male	57	33	24	
Female	35	13	22	
*BMI*				0.517
≤ 25	58	27	31	
> 25	34	19	15	
*Tumor size (cm)*				0.385
≤ 4	33	14	19	
> 4	59	32	27	
*Depths of invasion*				0.361
T1/T2	5	4	1	
T3/T4	87	42	45	
*Lymph node metastasis*				** < *0.001***
Negative	46	32	14	
Positive	46	14	32	
*Distal metastasis*				***0.019***
Negative	77	42	35	
Positive	15	4	11	
*TNM stage*				** < *0.001***
I/II	46	33	13	
III/IV	46	14	32	
*Histological type*				
Adenocarcinoma	76	38	38	0.881
Mucinous	7	3	4	
Others	9	5	4	
*Differentiation*				0.538
Well	1	1	0	
Moderate	70	34	36	
Poor	13	8	5	
Unknown	8	3	5	

Statistical significance was determined by Pearson χ^2^‐test, *P*-values in bold were statistically significant

**Table 2:** Univariate and multivariate analysis of overall survival in CRC patients

	Univariate			Multivariate		
Variables	HR	95% CI	*P*‐value	HR	95% CI	*P*‐value
Age (≤ 60 years vs. > 60 years)	0.601	0.265–1.359	0.221			
Gender (male vs. female)	0.507	0.231–1.111	0.09			
BMI (≤ 25 vs. > 25)	1.393	0.632–3.07	0.411			
Tumor size (≤ 4 cm vs. > 4 cm)	0.897	0.409–1.967	0.785			
Lymph node metastasis (N + vs. N0)	3.866	1.543–9.691	***0.004***	1.904	0.674–5.381	0.224
distal metastasis (M + vs M0)	5.187	2.311–11.64	** < *0.001***	3.971	1.659–9.506	***0.002***
TNM stage (III/IV vs. I/II)	1.063	1.02–1.109	***0.004***			
LOC101927668 expression (higher vs. lower)	3.742	1.493–9.377	***0.005***	2.782	1.036–7.471	***0.042***

*HR* hazard ratio, *CI* confidence interval. *P*-values were calculated using Cox proportional hazard regression analysis; those in bold indicated statistical significance

## LOC101927668 promoted CRC cell proliferation and metastasis in vitro

**Fig. 3:**
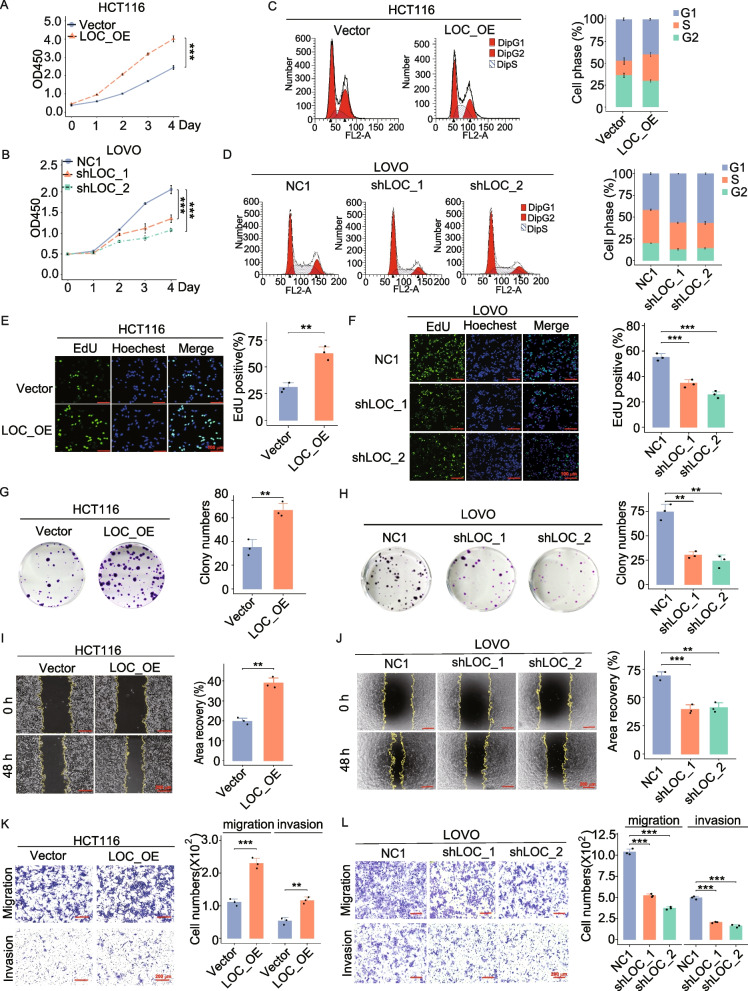
Impact of LOC101927668 on CRC cell proliferation and metastasis. **A**,** B** Assessment of cell proliferative ability through CCK8 in LOC101927668-overexpressing HCT116 cells or LOC101927668-depleted LOVO cells. **C**,** D** Analysis of cell cycle distribution by flow cytometry following LOC101927668 overexpression in HCT116 cells and LOC101927668 knockdown in LOVO cells. **E**,** F** EdU corporation assays were performed in LOC101927668 stably overexpressed or depleted CRC cells. **G**,** H** Colony formation assays were conducted to evaluate the reproductive capacity of CRC cells with overexpressed or silenced LOC101927668. **I-L** Evaluation of cell motility via wound healing assays (**I**,** J**) and transwell assays (**K, L**) subsequent to LOC101927668 overexpression in HCT116 cells and LOC101927668 depletion in LOVO cells. Data are presented as mean ± SD of at least three independent experiments. **P < 0.01, ***P < 0.001

To assess the biological functions of LOC101927668, the entire transcript (NCBI Refseq: NR_110114.1) was integrated into HCT116 and RKO cell genomes to establish CRC cells with stable overexpression of LOC101927668, achieved through vector construction, virus packaging, lentivirus infection, and puromycin selection. LOVO cells with stable downregulation of LOC101927668 were generated using shRNA introduction. Following the assessment of expression efficiencies in stably transfected CRC cells (Fig. S6), a series of assays including CCK8, flow cytometry, and EDU assays were conducted to evaluate the impact of LOC101927668 on CRC cell proliferation and cell cycle. As depicted in Fig. 3A and Fig. S7A-S7C, the overexpression of LOC101927668 promoted the proliferation of HCT116 and RKO cells, whereas its knockdown attenuated the growth capacity of LOVO cells. Cell cycle analysis indicated that the pro-proliferative impact of LOC101927668 on CRC cells was attributed, at least partially, to its facilitation of a higher proportion of cells entering the S phase (Fig. 3C and D, Fig. S7B). Stronger reproductive capacity was observed in cells with higher LOC101927668 expression, as examined by colony formation assay (Fig. 3G and H, Fig. S7D). Furthermore, accelerated migratory and invasive capabilities were observed in HCT116 and RKO cells overexpressing LOC101927668 compared to control cells, as evidenced by wound healing and transwell assays; conversely, LOC101927668-depleted LOVO cells exhibited the opposite effects (Fig.3I-3L, Fig. S7E).

## LOC101927668 interrupted p53 signaling by attenuating RBM47 expression

**Fig. 4:**
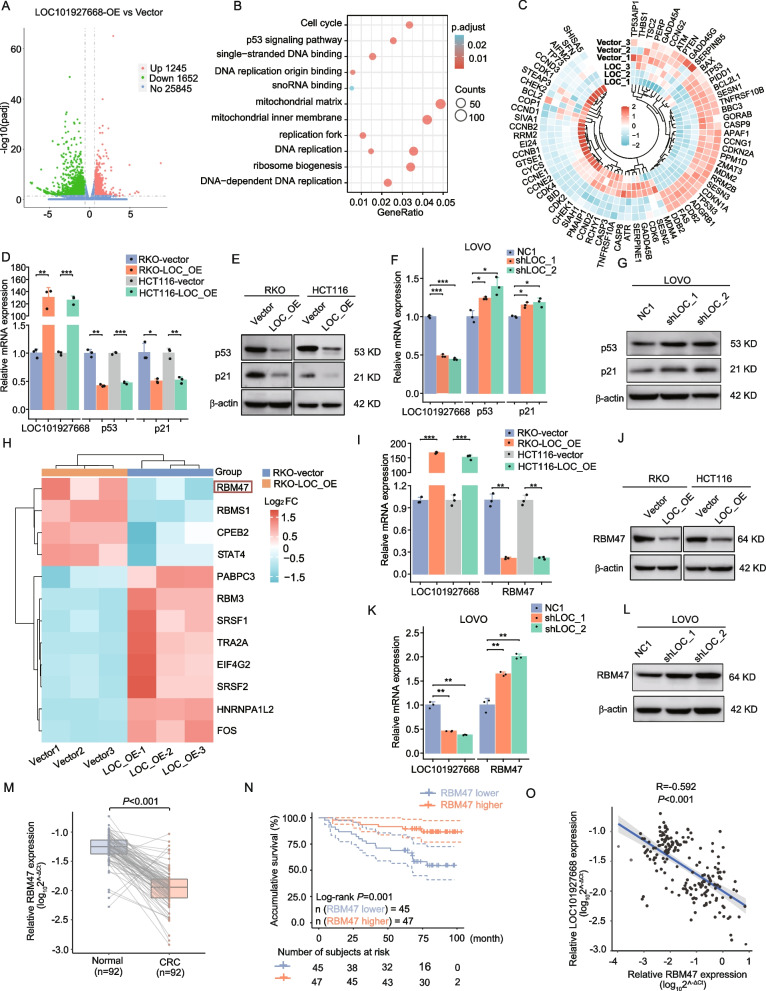
LOC101927668 dampens the p53 signaling pathway by downregulating RBM47 expression. **A** Volcano plot depicting the differential gene expression profile in LOC101927668-overexpressing RKO cells compared to control cells, as determined by RNA sequencing analysis. **B** KEGG analysis uncovering the enriched pathways among the differentially expressed genes (DEGs) in LOC101927668-overexpressing RKO cells in comparison to control cells. **C** Expression of genes involved in the p53 signaling pathway (KEGG hsa04115) in LOC101927668-overexpressing RKO cells compared to control cells, elucidated via mRNA profiling analysis. **D**,** E** Assessment of p53 and p21 expression levels via RT-qPCR (**D**) and Western blot (**E**) in RKO and HCT116 cells overexpressing LOC101927668, relative to their respective control cells. **F**,** G** Evaluation of p53 and p21 expression levels through RT-qPCR (**F**) and Western blot (**G**) analyses in LOVO cells depleted of LOC101927668, relative to their control counterparts. **H** Heatmap illustrating the expression levels of genes potentially regulating p53 in this study with |Fold change|≥ 2 and *P* < 0.05, as derived from RNA sequencing data. RBM47 was highlighted with a red box. **I**,** J** Analysis of RBM47 expression through RT-qPCR (**I**) and Western blot (**J**) subsequent to LOC101927668 overexpression in RKO and HCT116 cells. **K**,** L** Assessment of RBM47 expression levels via RT-qPCR (**K**) and Western blot (**L**) following LOC101927668 knockdown in LOVO cells. M. RBM47 expression in 92 paired CRC tissues and corresponding adjacent normal tissues analyzed by RT-qPCR. N. Kaplan-Meier analysis of overall survival in 92 CRC patients stratified by RBM47 expression. O. The correlation between LOC101927668 and RBM47 expression in 92 CRC patients assessed through Pearson correlation analysis. Data are presented as mean ± SD of at least three independent experiments. *P < 0.05, **P < 0.01, ***P < 0.001

To elucidate the underlying mechanism by which LOC101927668 promoted colorectal cancer progression, RNA sequencing was employed to identify differentially expressed genes in LOC101927668-overexpressed RKO cells compared to control cells. Overexpression of LOC101927668 resulted in the upregulation of 1245 genes and the downregulation of 1652 genes (|FC|≥ 1.5, *P* < 0.05, Fig. 4A). Functional enrichment analysis of these genes using KEGG and GO analyses revealed a significant impact of LOC101927668 on the p53 signaling pathway (Fig. 4B). The expression of all genes in the p53 signaling pathway (KEGG entry: hsa04115) was extracted and visualized using a Circos heatmap to elucidate the impact of LOC101927668 on p53 signaling (Fig. 4C). Subsequent qPCR validation in RKO and HCT116 cells with enforced expression of LOC101927668 confirmed these findings (Fig. S8). The mRNA and protein levels of p53 and p21 (also named as CDKN1A), two key components of the p53 pathway, were significantly downregulated in RKO and HCT116 cells with elevated LOC101927668, and upregulated in LOC101927668-silenced LOVO cells (Fig. 4D-G).

Since p53 transcript abundance could be regulated at both the transcriptional and post-transcriptional levels, factors involved in p53 transcriptional regulation or post-transcriptional modification may serve as intermediates in LOC101927668-mediated changes in p53 expression. Based on this assumption, upregulation of LOC101927668 would alter the expression of these intermediate factors, subsequently affecting p53 mRNA expression. Therefore, well-known p53 transcription factors such as AP1, NF-κB, HOXA5, and RBM47 [11, 46], as well as proteins involved in p53 alternative splicing or mRNA stability, such as SRSF3 and CPEB2 [47, 48], were considered as potential intermediates. Additionally, predicted p53 transcription factors and mRNA-binding proteins identified through online bioinformatics tools (PROMO [49] and RBPmap [50]) were also included as candidates. The expression of these candidates was examined in RNA sequencing data of LOC101927668-overexpressing RKO cells compared to control cells. Among the genes showing significant expression differences (|FC|≥ 2, *P* < 0.05), RBM47, a validated p53 transcription factor [11], was identified (Fig. 4H). Subsequent qPCR and Western blot analyses confirmed that LOC101927668 overexpression significantly suppressed RBM47 expression, while its knockdown increased RBM47 expression at both mRNA and protein levels in CRC cells (Fig. 4I-L). Additionally, RBM47 expression was notably lower in CRC specimens compared to paired noncancerous tissues in our cohort (Fig. 4M), as well as in TCGA and GEO datasets (Fig. S9A-S9E). Reduced RBM47 expression was significantly associated with poorer survival in both our cohort and GSE40967 (Fig. 4N, Fig. S9F). A significant negative correlation between LOC101927668 and RBM47 was observed not only in our paired tissue samples but also in several GEO datasets (Fig. 4O, Fig. S9G-S9K). The elevated LOC101927668 expression in patients with chr7p21.1 amplification, coupled with its negative correlation with both RBM47 and p53 in tissue samples from nine CRC patients examined by qPCR, further supported the existence of a regulatory axis involving CNV-LOC101927668-RBM47-p53 in vivo (Fig. S10).

## LOC101927668 regulated RBM47 expression by modulating its mRNA stability

**Fig. 5:**
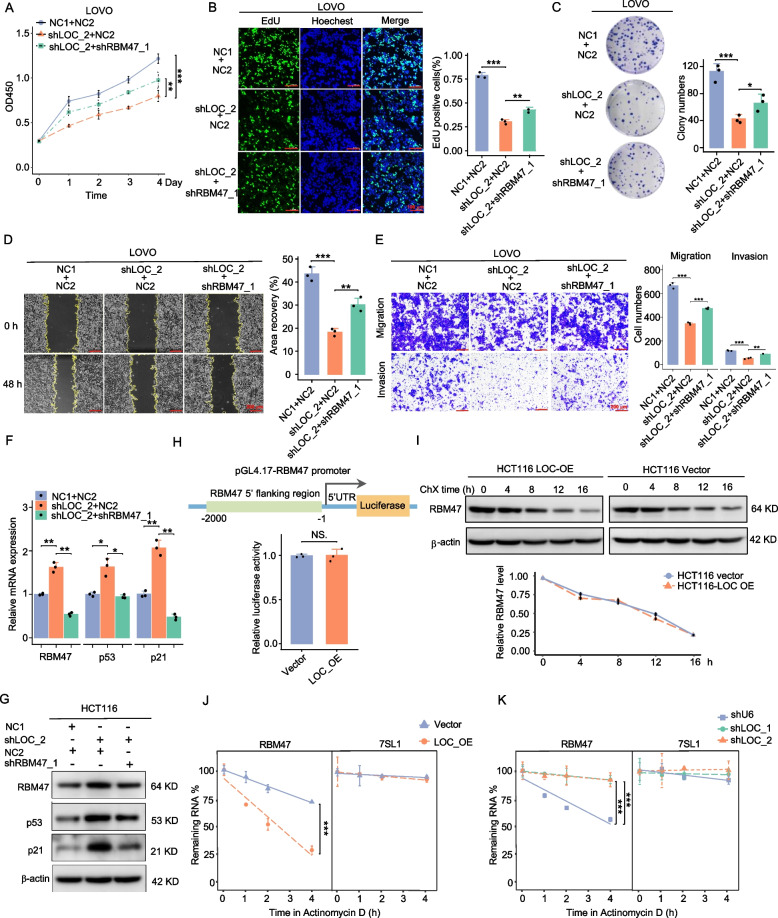
Regulation of RBM47 expression by LOC101927668 through modulation of mRNA stability. **A-C** Cell proliferative ability was assessed in LOVO cells with or without RBM47 knockdown upon LOC101927668 depletion using CCK8 (**A**), EdU (**B**), and colony formation assays (**C**). **D**,** E** Evaluation of cell motility via wound healing assay (**D**) and transwell assay (**E**) in LOC101927668-depleted LOVO cells with or without RBM47 knockdown. **F-G** Evaluation of RBM47, p53, and p21 expression levels using RT-qPCR (**F**) and Western blot (**G**) in LOC101927668-depleted LOVO cells with or without RBM47 knockdown. **H** Construction of the RBM47 promoter luciferase reporter plasmid (upper half), and luciferase activity detection in HEK293T cells co-transfected with pGL4.17-RBM47 promoter plasmid and LOC101927668 overexpression or a control vector (lower half). **I** HCT116 cells overexpressing LOC101927668 and their control counterparts were treated with cycloheximide (CHX) for the specified duration. RBM47 levels were subsequently measured by Western blot analysis. **J**,** K** The mRNA stability of RBM47 in LOC101927668-overexpressing HCT116 cells (**J**) and LOC101927668-knockdown LOVO cells (**K**) was quantified by RT-qPCR after treatment with actinomycin D for the indicated durations, groups with statistical significance were presented using asterisk. Data are presented as mean ± SD of at least three independent experiments. *P < 0.05, **P < 0.01, ***P < 0.001

To investigate whether the proliferative and metastatic effects induced by ectopically overexpressed LOC101927668 were dependent upon RBM47/p53 signaling, LOVO cells with depleted LOC101927668 were transfected with a plasmid containing effective RBM47 shRNA (shRBM47_1). The expression efficiency of RBM47 shRNAs was validated prior to experimentation (Fig. S11). Rescue assays, encompassing cell viability assessments using CCK8, EDU incorporation analysis, and colony formation assay, demonstrated that the attenuated pro-proliferative effect resulting from reduced LOC101927668 expression was partially restored upon RBM47 depletion (Fig. 5A-C). Furthermore, the attenuated cell motility observed following LOC101927668 knockdown was partially rescued upon downregulation of RBM47, as demonstrated by wound healing and transwell assays (Fig. 5D and E). Additionally, the upregulated expression of p53 and p21 resulting from LOC101927668 knockdown was attenuated following RBM47 depletion, observed at both the mRNA and protein levels (Fig. 5F and G). These findings suggest that LOC101927668 may exert pro-tumorigenic roles, at least in part, through the inactivation of the RBM47/p53 signaling pathway.

To elucidate the regulatory mechanism of LOC101927668 on RBM47 expression, we initially assessed the impact of LOC101927668 on RBM47 promoter activity. Nearly unchanged luciferase activity suggested that LOC101927668 modulated RBM47 expression at the post-transcriptional level (Fig. 5H). Subsequently, we observed a similar degradation rate of RBM47 protein in the presence or absence of LOC101927668 overexpression, by employing cycloheximide (ChX) to inhibit protein synthesis (Fig. 5I). To further explore the influence of LOC101927668 on RBM47 mRNA stability, we blocked new RNA synthesis using actinomycin D in CRC cells overexpressing or silencing LOC101927668. Compared with the control group, the degradation rate of RBM47 mRNA was significantly accelerated when LOC101927668 was upregulated and decelerated when LOC101927668 was silenced, suggesting that LOC101927668 regulated the level of RBM47 by modulating its mRNA stability. RN7SL1 was detected as a negative control (Fig. 5J and K).

## The interaction between LOC101927668 and hnRNPD resulted in the decreased mRNA stability of RBM47

**Fig. 6:**
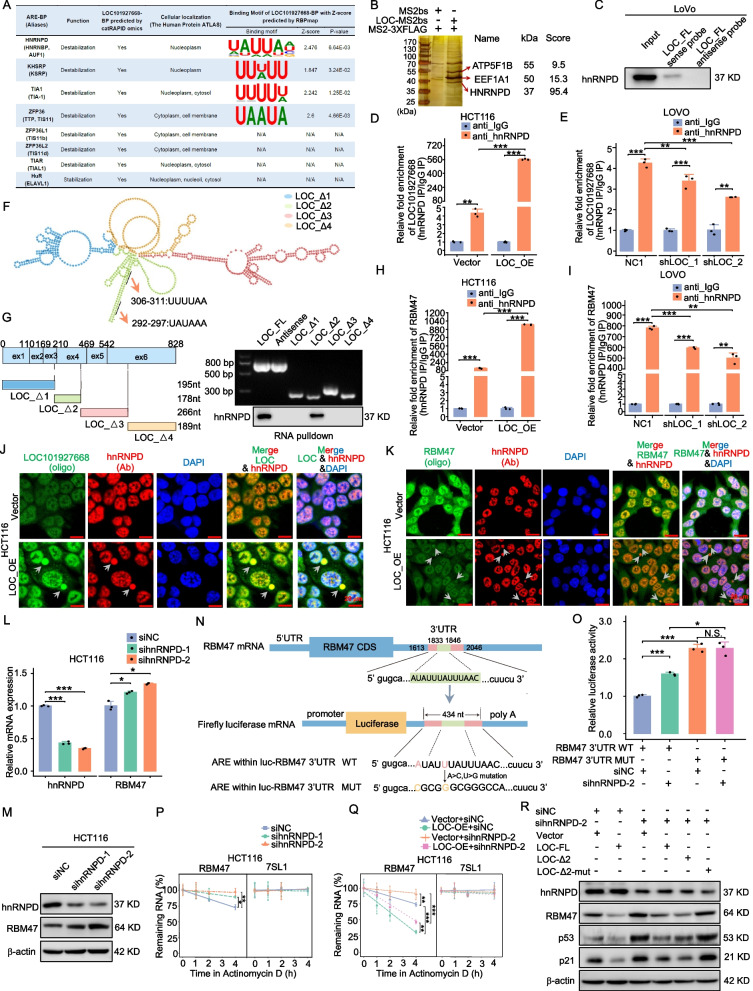
The interaction between LOC101927668 and hnRNPD led to a reduction in the mRNA stability of RBM47. **A** The screening and ranking of ARE-binding proteins interacting with LOC101927668 were performed through integrative analysis utilizing several bioinformatic tools. **B** Protein complexes obtained from the in vivo RNA pull-down assay were separated by SDS-PAGE and visualized using silver staining. Specific bands were excised and analyzed via mass spectrometry, with the top-ranking proteins presented along with their respective scores. **C** In vitro RNA pull-down assays were conducted using either sense or antisense probes specific to LOC101927668, the level of hnRNPD was detected via Western blot subsequently. **D**,** E** The interaction between endogenous LOC101927668 and hnRNPD was assessed in LOC101927668-overexpressing HCT116 cells (**D**) and LOC101927668-knockdown LOVO cells (**E**) using RIP followed by RT-qPCR analysis. **F** LOC101927668 was segmented into four parts based on its secondary structure. The predicted binding sites between LOC101927668 and hnRNPD were identified within the second truncated fragment of LOC101927668. **G** Left panel: Schematic representation of truncated LOC101927668. Right upper panel: Confirmation of fragment sizes of full-length LOC101927668 (FL), antisense-LOC101927668, and various truncated isoforms via PCR. Right lower panel: Immunoblotting of hnRNPD in RNA pull-down extracts by using the LOC101927668 fragments mentioned above. **H**,** I** The interaction between endogenous RBM47 and hnRNPD was evaluated in LOC101927668-overexpressing HCT116 cells (**H**) and LOC101927668-knockdown LOVO cells (**I**) using RIP followed by RT-qPCR analysis. **J**,** K** LOC101927668 ISH combined with hnRNPD IF (**J**), and RBM47 RNA ISH alongside hnRNPD IF (**K**) were conducted in HCT116 cells with and without LOC101927668 overexpression. Grey arrows indicating specific RNA–protein complexes. **L**,** M** The expression levels of RBM47 mRNA and protein were assessed via RT-qPCR (**L**) and Western blot (**M**) subsequent to transfection of HCT116 cells with hnRNPD siRNA. **N** Schematic depiction of pGL3-control plasmids containing either the wild-type or mutated AU-rich element (ARE) sequence from the RBM47 3’UTR. **O** The dual luciferase reporter assay was utilized to evaluate the luciferase activity of HCT116 cells transfected with the indicated reporter plasmids and hnRNPD siRNA. **P** The mRNA stability of RBM47 in HCT116 cells treated with hnRNPD siRNA was quantified by RT-qPCR after treatment with actinomycin D for the indicated durations. RN7SL1 was used as a negative control. **Q** The mRNA stability of RBM47 in HCT116 cells with or without hnRNPD knockdown upon LOC101927668 overexpression was quantified using RT-qPCR following treatment with actinomycin D for the specified durations. RN7SL1 was used as a negative control. **R** RBM47, p53, and p21 protein levels were assessed via Western blot in cells with or without hnRNPD siRNA, following overexpression of full-length (FL), the second truncated fragment (Δ2), as well as its mutated form of LOC101927668 (Δ2-mut). Data are presented as mean ± SD of at least three independent experiments. *P < 0.05, **P < 0.01, ***P < 0.001

To facilitate RBM47 destruction, LOC101927668 was hypothesized to bind to a protein with RNA degradation capacity. This protein might translocate from the nucleus to the cytoplasm upon interaction with LOC101927668, leading to the degradation of RBM47 mRNA. By employing bioinformatic analysis of the RBM47 3' untranslated region (3'UTR) with the ARED-Plus database [51], a sequence containing AU-rich elements (AREs) was identified within the RBM47 transcript's 3'UTR. These AREs are recognized as targets for mRNA stabilization or degradation by ARE-binding proteins (ARE-BPs) [51–53]. By using the catRAPID omics tool to predict LOC101927668 RNA-binding proteins [54], eight established ARE-BPs potentially interacting with LOC101927668 were identified (Fig. 6A). After thorough analysis of their function, intracellular localization, and potential binding motifs, several ARE-BPs meeting the selection criteria were identified (Fig. 6A). HnRNPD, an ARE-BP primarily expressed in the nucleoplasm, capable of translocating to the cytoplasm to destabilize mRNA [55], was subsequently examined due to its highest z-score and minimum *P* value of potential binding motifs (Fig. 6A).

Thein vivo RNA–protein pull-down assay followed by mass spectrometry confirmed our inference. The top-ranking protein for each band was identified, with hnRNPD receiving the highest score in the ~ 37 kDa band (Fig. 6B). The in vitro RNA pull-down assay followed by Western blot analysis additionally validated the direct interaction between LOC101927668 and hnRNPD (Fig. 6C). The RIP assay was further conducted to enrich all RNAs binding with hnRNPD in HCT116 cells overexpressing LOC101927668 and LOVO cells with depleted LOC101927668. Following reverse transcription, the cDNA was subjected to qPCR to detect LOC101927668 expression subsequently. Compared to the nonspecific anti-IgG group, the anti-hnRNPD group showed a significant increase in LOC101927668 levels, with the quantity of LOC101927668 positively correlated with its cellular abundance (Fig. 6D and E). This reaffirmed the association between hnRNPD and LOC101927668. According to the spatial secondary structure of LOC101927668 and its predicted binding motifs with hnRNPD, the LOC101927668 sequence was segmented into four fragments for truncated expression clone construction, each distinguished by a different color. The anticipated binding sites were recognized at 292–297 and 306–311 bp of the second truncated fragment (Fig. 6F). Subsequent RNA pull-down assays confirmed the binding of hnRNPD with LOC101927668 within the predicted domain spanning 196–373 bp (Fig. 6G).

Furthermore, the direct interaction between LOC101927668 and RBM47 was confirmed through RIP assays, where RBM47 expression significantly changed in the anti-hnRNPD group, correlating with LOC101927668 expression in cells with either overexpressed or depleted LOC101927668, compared to the control group (Fig. 6H and I). The results from LOC101927668 FISH and hnRNPD IF, in combination with RBM47 RNA FISH and hnRNPD IF, indicated that in HCT116 cells with normal LOC101927668 expression, hnRNPD was situated in the nucleus, and LOC101927668 was predominantly nuclear with some presence in the cytoplasm. While in cells overexpressing LOC101927668, a portion of both LOC101927668 and hnRNPD were observed to shift into the cytoplasm (Fig. 6J). In control groups, approximately 2.3% of cells exhibited translocation of the LOC101927668-hnRNPD complex from the nucleus to the cytoplasm, while this proportion increased to nearly 15% when LOC101927668 was overexpressed (Fig. S12A). Additionally, the probe intensity of RBM47 mRNA decreased significantly with LOC101927668 upregulation, along with an increased proportion of cells showing co-localization of RBM47 mRNA and hnRNPD in the cytoplasm (Fig. 6K and Fig. S12B). These findings suggest that overexpressed LOC101927668 binds to hnRNPD, triggering its translocation from the nucleus to the cytoplasm, where hnRNPD facilitates the decay of RBM47 mRNA.

In addition, hnRNPD knockdown using siRNA significantly increased the mRNA and protein expression of RBM47 (Fig. 6L and M). To further investigate whether hnRNPD was bound to the ARE domain of RBM47, potentially leading to RBM47 transcript decay, a dual-luciferase reporter assay was conducted. The predicted RBM47 3'UTR ARE site, identified using the ARED-Plus database [51], along with two 200 bp flanking sequences at both ends, was inserted into the 3'UTR region of the PGL3-control plasmid. Additionally, a corresponding mutation plasmid was generated based on the principles of A > C and U > G substitutions (Fig. 6N). The luciferase activity of the vector carrying wild-type RBM47 3'UTR (WT) was lower than that of the mutant type (MUT). Upon hnRNPD siRNA treatment, a partial restoration of luciferase activity of RBM47 3'UTR WT was observed, while no change was detected for RBM47 3'UTR MUT (Fig. 6O). Nuclear/cytoplasmic protein separation and extraction revealed hnRNPD's predominant localization in the nucleus of CRC cells, while RBM47 was detected in both the nucleus and cytoplasm. Ectopic overexpression of LOC101927668 led to decreased RBM47 expression in both nuclear and cytoplasmic compartments, with a slight reduction in hnRNPD levels within the nucleus (Fig. S12C). Moreover, RNA stability testing showed that hnRNPD depletion decelerated RBM47 mRNA degradation (Fig. 6P). SihnRNPD-2, selected for the subsequent rescue experiment due to its superior knockdown efficiency, partially reversed the accelerated degradation of RBM47 caused by overexpressed LOC101927668 (Fig. 6Q). Subsequent Western blot analysis confirmed that hnRNPD knockdown partially restored the attenuated RBM47/p53 signaling pathway resulting from ectopic overexpression of LOC101927668. Importantly, this rescue effect depended on the interaction between hnRNPD and the second fragment of LOC101927668 containing the binding domains (Fig. 6R).

## LOC101927668 facilitated CRC cell proliferation and metastasis by regulating RBM47 in vivo

**Fig. 7:**
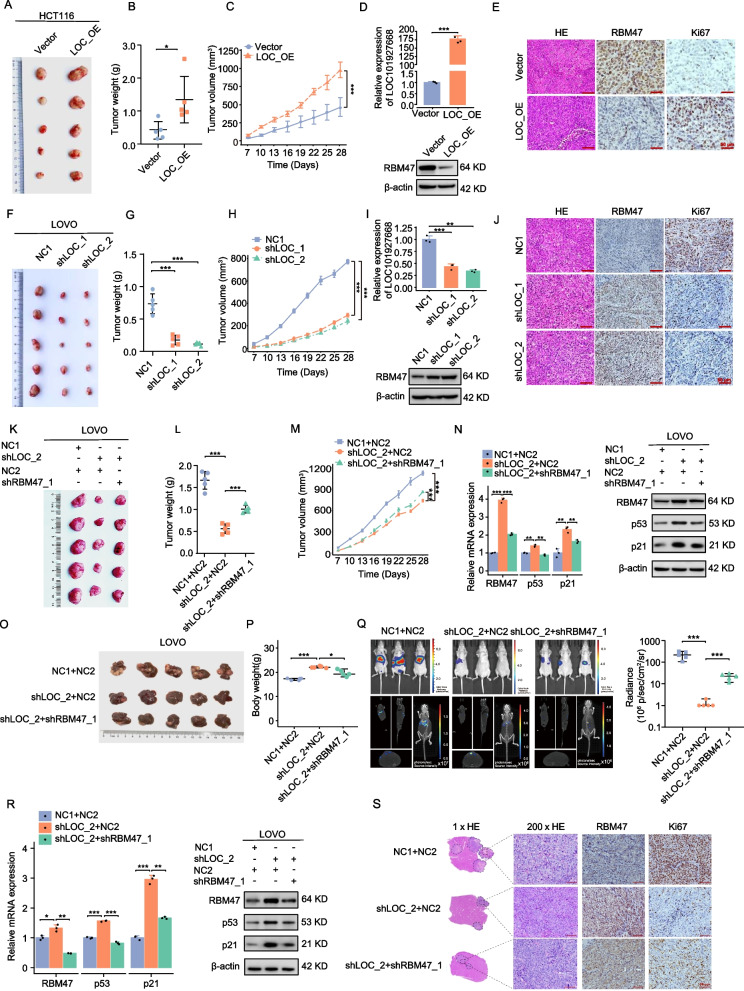
LOC101927668 played a crucial role in promoting CRC cell proliferation and metastasis by targeting RBM47 in vivo. **A-E** Subcutaneous implantation model of HCT116 cells stably overexpressing LOC101927668 or its control vector. (**A**) Macroscopic appearance of tumors and (**B**) tumor weight analysis upon sacrifice of the mice. (**C**) Tumor growth curves recorded during the experiment. (**D**) Analysis of LOC101927668 and RBM47 expression in tumor tissues via RT-qPCR and Western blot. (**E**) HE sections and representative IHC staining of RBM47 and Ki67 in different groups of tumor tissues. **F-J** Subcutaneous implantation model of LOVO cells with stable LOC101927668 depletion. (**F**) Macroscopic appearance of tumors, (**G**) tumor weight analysis, and (**H**) growth curves were presented. (**I**) Analysis of LOC101927668 and RBM47 expression via RT-qPCR and Western blot, alongside (**J**) histological examination and representative IHC staining of RBM47 and Ki67. **K**-**N** Subcutaneous implantation model of LOVO cells with stable LOC101927668 depletion and RBM47 knockdown. (**K**) Macroscopic appearance of tumors, (L) tumor weight analysis, and (M) growth curves were shown. (**N**) Expression of LOC101927668 and important molecules in RBM47/p53 signaling were detected in tumor tissues via RT-qPCR and Western blot. O-S. Orthotopic liver xenograft tumor model of LOVO cells with stable depletion of LOC101927668 and knockdown of RBM47. Macroscopic images depicting the morphological changes in mouse livers (**O**) and body weight of mice (**P**) upon sacrifice. (**Q**) Representative two-dimensional (2D) and three-dimensional (3D) bioluminescence imaging of mice with the quantification of two-dimensional bioluminescence imaging. (**R**) Detection of LOC101927668 and important molecules in RBM47/p53 signaling via RT-qPCR and Western blot. (**S**) HE sections and representative IHC staining of RBM47 and Ki67 in different groups of liver metastatic tissues. Data are presented as mean ± SD of at least three independent experiments. *P < 0.05, **P < 0.01, ***P < 0.001

To investigate the role of LOC101927668 in tumorigenesis in vivo, we conducted subcutaneous implantation of HCT116 cells stably overexpressing LOC101927668 and LOVO cells with stable depletion of LOC101927668 in nude mice. The results revealed that, compared with the corresponding control group, mice bearing enhanced LOC101927668 demonstrated significantly accelerated tumor growth (Fig. 7A-C), while tumors in mice with depleted LOC101927668 were significantly reduced (Fig. 7F-H). The overexpression or knockdown efficiency of LOC101927668 was confirmed in tumor samples from mouse xenograft models through RT-qPCR, accompanied by corresponding changes in RBM47 expression by Western blot analyses (Fig. 7D and I). Furthermore, IHC staining provided additional verification of the impact of LOC101927668 on tumor proliferation by detecting Ki67, as well as its suppressive effect on RBM47 expression (Fig. 7E and J).

Subsequently, we employed both a subcutaneous implantation model and an orthotopic hepatic metastatic xenograft mouse model by splenic injection to investigate the influence of LOC101927668 and RBM47 on the development and metastasis of CRC. In the subcutaneous tumor model, the attenuated tumor growth resulting from LOC101927668 knockdown was partially restored upon depletion of RBM47 (Fig. 7K). Expression of RBM47, p53 and p21 was assessed through RT-qPCR and Western blot in relevant groups (Fig. 7N). Consistent with the findings in subcutaneous tumors, the attenuation of tumor metastasis due to downregulated LOC101927668 expression could be partially reversed by decreased RBM47 expression. This was confirmed by luminescence observation of mouse livers and liver tissues harvested upon sacrifice (Fig. 7O), as well as by assessing the expression of key molecules in the RBM47/p53 signaling pathway via qPCR and Western blot (Fig. 7R). Histological assessment, utilizing HE staining and IHC analysis of liver metastasis samples, revealed notable findings. Specifically, knockdown of LOC101927668 attenuated the metastatic potential of LOVO cells, whereas decreased expression of RBM47 exacerbated the formation of metastatic nodules, resulting in increased size and number. The proliferative efficiency of metastatic nodules, as indicated by Ki67 staining, exhibited an inverse correlation with RBM47 expression across the analyzed groups (Fig. 7S).

## Discussion

CNV, a hallmark of genome instability, is pivotal in cancer pathogenesis, with amplified genomic regions often harboring oncogenic genes, while deleted segments frequently encompass tumor suppressor genes [56, 57]. In our cohort, the CNVs identified in nine individuals with CRC closely resembled previous research findings [19, 58]. Amplification events were notably frequent in chromosomal regions 7p, 8, 12p, 20, and Xp, while deletion events predominated in regions such as chromosomes 3, 5q, 8p, 14q, 15q, 16p, 17p, 18q, and 22q. It is noteworthy that 4.8–9.5% of the human genome is comprised of CNVs [59], whereas protein-coding genes constitute only 2% of the entire human genome [60]. Given that up to 90% of the human genome is transcriptionally active [61], it is conceivable that noncoding RNAs, harboring significant tumorigenic functionalities, may originate from regions altered by CNVs. Indeed, aberrant CNV-induced lncRNAs exert significant influences on tumor development [18]. For example, MAT1, a CNV-associated lncRNA, drives uveal melanoma by inhibiting the interaction between the MLL protein complex and the tumor suppressor PCDH20 promoter. This disrupts H3K4 trimethylation, impairing PCDH20 transcription [62]. Although previous studies have elucidated the functions of numerous dysregulated lncRNAs influenced by CNVs, there remains a significant gap in our understanding of CNV-associated lncRNAs.

Based on our analytical pipeline, we identified LOC101927668, which exhibited significant overexpression attributed to copy number gain of chr7p21.1 in CRC tissues, as the focus of our current investigation. Moreover, LOC101927668 was found overexpressed in other malignancies as well, such as lung cancer, pancreatic cancer, and papillary renal cell carcinoma (Fig. S3). Chromosome 7 amplification has also been reported in these tumors [63–65]. This consistent overexpression and copy number amplification across various cancer types suggest that SCNA-induced LOC101927668 overexpression may play a broader oncogenic role beyond CRC. Additionally, LOC101927668 overexpression in CRC patients without 7p21.1 amplification indicates that its upregulation is not solely due to copy number amplification; transcriptional regulation likely plays a significant role as well. Since LOC101927668 expression remained consistent in CRC patients regardless of KRAS and BRAF mutation status (Fig. S13A), we investigated several transcription factors, including MACC1, E2F3, and SIX2, known for their oncogenic roles in CRC tumorigenesis [66–68]. These factors were not only highly expressed in CRC tissues compared to adjacent normal tissues but also exhibited a strong correlation with LOC101927668 expression (Fig. S13B-S13D). Further studies are required to unravel the underlying mechanisms.

HnRNPD, also known as AU-binding factor 1 (AUF1), serves as an ARE-BP pivotal in governing mRNA degradation processes. As the inaugural member of the translation and turnover regulatory (TTR)-RBP family, hnRNPD exhibits dynamic intracellular localization, shuttling between the nucleus and cytoplasm [69, 70]. Its selective affinity for specific target transcripts can vary across different cellular contexts and tissue types [55, 71]. HnRNPD is a multifunctional protein exerting regulatory influence on diverse nuclear processes, encompassing telomere maintenance, transcriptional activation, and alternative splicing [71, 72]. In the cytoplasm, hnRNPD selectively binds to AU-rich sequences within mRNA molecules. Its impact on downstream target transcripts can either facilitate mRNA decay or augment their stability and translation [55]. Prior research has elucidated that lncRNAs can modulate the mRNA stability of specific target genes by interacting with hnRNPD, thereby exerting a notable influence on tumor initiation and progression. Linc01354 has been shown to activate the Wnt/β-catenin signaling pathway by stabilizing β-catenin mRNA through its interaction with hnRNPD, consequently promoting the proliferation and metastasis of CRC cells [73]. Additionally, a separate investigation revealed that the interaction between lncRNA THOR and hnRNPD led to hnRNPD stabilization and activation of the PI3K/AKT pathway, thereby facilitating the progression of breast cancer [74].

In interphase cells, both LOC101927668 RNA and hnRNPD protein were primarily localized in the nucleus, with RBM47 nascent and mature mRNA distributed in nucleus and cytoplasm. While during mitosis, LOC101927668, hnRNPD, and RBM47 were all translocated to the cytoplasm (Fig. S14). It is well known that during interphase, hundreds to thousands of coding and noncoding RNAs are retained in the nucleus to regulate chromatin structure and gene expression [75]. However, during cell division, nuclear RNA transcription is significantly reduced, and many nascent transcripts and components of the transcription machinery are removed from mitotic chromosomes to facilitate chromosome segregation into daughter cells [76]. As shown in Fig. S14, no matter the sublocation of LOC101927668 and RBM47 in interphase, they became diffusely distributed in the cytoplasm during mitosis.

Additionally, previous studies have reported that hnRNP proteins disperse throughout the cell when the nuclear envelope breaks down in mitosis, but return to the newly formed nuclei as the nuclear envelope of the daughter cells is assembled [77, 78]. Therefore, we hypothesize that LOC101927668-induced attenuation of RBM47/p53/p21 signaling through hnRNPD recruitment primarily occurs during interphase. The translocation of hnRNPD, followed by the degradation of RBM47 mRNA, is largely stimulated by overexpressed LOC101927668. Throughout mitosis, LOC101927668 and hnRNPD are detatched from mitotic chromatin and relocated to the cytoplasm, later returning to the newly formed nuclei to resume their regulatory activities after mitosis.

**Fig. 8:**
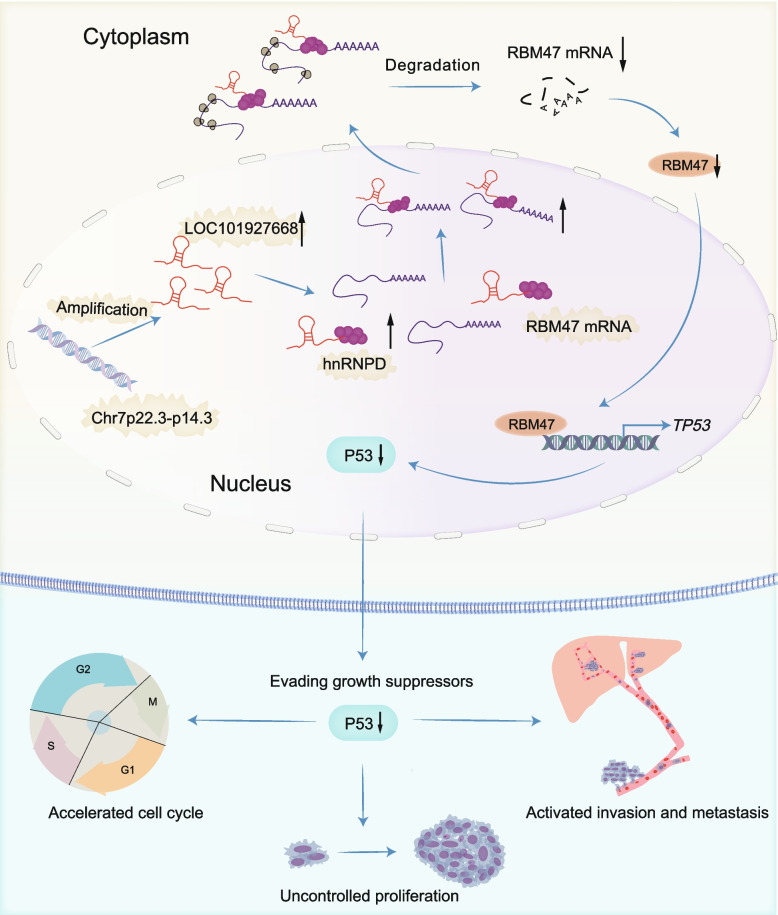
Schematic representation illustrating the pathway by which LOC101927668 recruits hnRNPD protein to modulate the p53 signaling axis via facilitating the degradation of RBM47 mRNA, ultimately promoting CRC progression

Our findings reveal a novel mechanism in which copy number amplification-induced overexpression of LOC101927668 interacts with hnRNPD, leading to their translocation to the cytoplasm. In the cytoplasm, hnRNPD binds to the ARE motif within the 3' UTR of RBM47 mRNA, destabilizing it and ultimately disrupting the p53 signaling pathway (Fig. 8). Given its widespread overexpression and potential role in promoting tumor growth, LOC101927668 presents itself as a promising therapeutic target. Inhibiting LOC101927668 could suppress tumor progression and enhance the efficacy of existing treatments. Clinical strategies might involve the development of small molecule inhibitors, antisense oligonucleotides, or RNA interference technologies specifically designed to target LOC101927668. Future research should focus on understanding the precise mechanisms by which LOC101927668 contributes to tumorigenesis in different cancer types, as well as on the development and testing of potential therapeutic agents targeting this lncRNA in clinical settings.

## Conclusions

In conclusion, our study proposes that dysregulation of LOC101927668, induced by CNV, promotes the progression of CRC by interacting with hnRNPD. This interaction inhibits RBM47 expression at the post-transcriptional level, consequently attenuating the RBM47/p53/p21 signaling axis (Fig. 8). This study not only elucidates the mechanism underlying RBM47 inactivation but also underscores a promising therapeutic target for cancer treatment. Inhibition of the elevated expression of LOC101927668 to restore RBM47 activity represents a distinctive and promising therapeutic strategy.

## Supplementary Information

Supplementary Material 1.

## Data Availability

RNA sequencing data were deposited into the Gene Expression Omnibus database under accession number GSE268122 and are available at the following URL: https://www.ncbi.nlm.nih.gov/geo/query/acc.cgi?&acc=GSE268122.

## Abbreviations

SCNAs: Somatic copy number alterations
lncRNAs: Long non-coding RNAs
CRC: Colorectal cancer
AREs: AU-rich elements
ARE-BPs: AU-rich element binding proteins
UTR: Untranslated region
TCGA: The Cancer Genome Atlas
GEO: Gene Expression Omnibus
RBPs: RNA-binding proteins
CNVs: Copy number variations
HnRNPD: Heterogeneous nuclear ribonucleoprotein D
ATCC: American Type Culture Collection
RPMI 1640: Roswell Park Memorial Institute 1640
DMEM: Dulbecco’s Modified Eagle Medium
FBS: Fetal bovine serum
STR: Short tandem repeat
ISH: In situ hybridization
FISH: Fluorescence in situ hybridization
shRNA: Short hairpin RNA
LOC-FL: Full-length LOC101927668
WT: Wild-type
Mut: Mutation
RT-qPCR: Reverse transcription-quantitative PCR
RIP: RNA immunoprecipitation
CHX: Cycloheximide
IHC: Immunohistochemistry
GO: Gene Ontology
KEGG: Kyoto Encyclopedia of Genes and Genomes
FC: Fold change
