# Correction: Copy number amplification-induced overexpression of lncRNA LOC101927668 facilitates colorectal cancer progression by recruiting hnRNPD to disrupt RBM47/p53/p21 signaling

**DOI:** 10.1186/s13046-026-03772-w

**Published:** 2026-07-01

**Authors:** Zaozao Wang, Haibo Han, Chenghai Zhang, Chenxin Wu, Jiabo Di, Pu Xing, Xiaowen Qiao, Kai Weng, Hao Hao, Xinying Yang, Yifan Hou, Beihai Jiang, Xiangqian Su

**Affiliations:** 1 Key Laboratory of Carcinogenesis and Translational Research (Ministry of Education/Beijing), Department of Gastrointestinal Surgery IV, Peking University Cancer Hospital & Institute, No.52 Fucheng Road, Haidian District, Beijing, 100142 China; 2 Key Laboratory of Carcinogenesis and Translational Research (Ministry of Education/Beijing), Department of Clinical Laboratory, Peking University Cancer Hospital and Institute, No.52 Fucheng Road, Haidian District, Beijing, 100142 China; 3 State Key Laboratory of Holistic Integrative Management of Gastrointestinal Cancers, Beijing Key Laboratory of Carcinogenesis and Translational Research, Department of Gastrointestinal Surgery IV, Peking University Cancer Hospital & Institute, No.52 Fucheng Road, Haidian District, Beijing, 100142 China

**Correction: J Exp Clin Cancer Res 43, 274 (2024)**

**https://doi.org/10.1186/s13046-024-03193-7**

Following the publication of the original article [1], the authors identified errors in Fig. 3L. The representative Transwell invasion images, an image from the shLOC2 group (from the migration assay) was mistakenly inserted in place of the correct image for the NC1 group (from the invasion assay).

Incorrect Fig.  3L

**Fig. 3:**
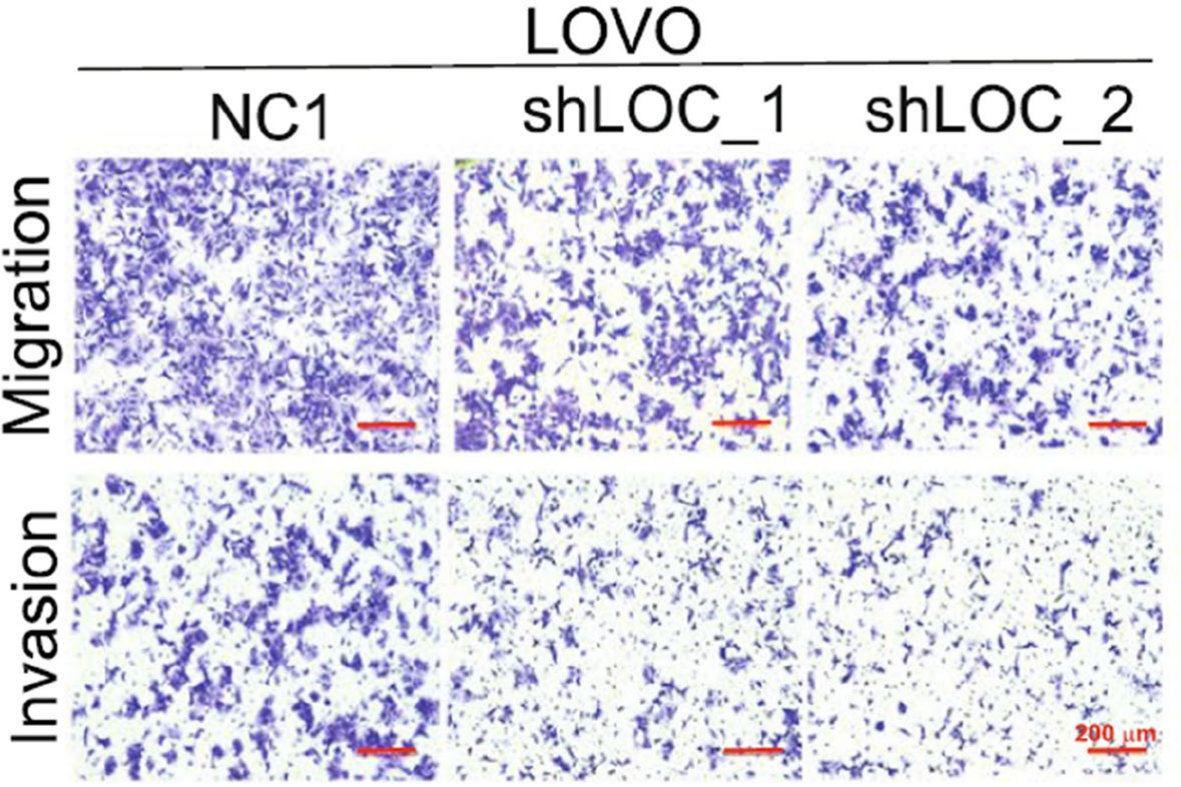
Impact of LOC101927668 on CRC cell proliferation and metastasis. **A**, **B** Assessment of cell proliferative ability through CCK8 in LOC101927668-overexpressing HCT116 cells or LOC101927668-depleted LOVO cells. **C**, **D** Analysis of cell cycle distribution by flow cytometry following LOC101927668 overexpression in HCT116 cells and LOC101927668 knockdown in LOVO cells. **E**, **F** EdU corporation assays were performed in LOC101927668 stably overexpressed or depleted CRC cells. **G**, **H** Colony formation assays were conducted to evaluate the reproductive capacity of CRC cells with overexpressed or silenced LOC101927668. **I**-**L** Evaluation of cell motility via wound healing assays (**I**, **J**) and transwell assays (**K**, **L**) subsequent to LOC101927668 overexpression in HCT116 cells and LOC101927668 depletion in LOVO cells. Data are presented as mean ± SD of at least three independent experiments. **P < 0.01, ***P < 0.001

Correct Fig. 3L

**Fig. 3:**
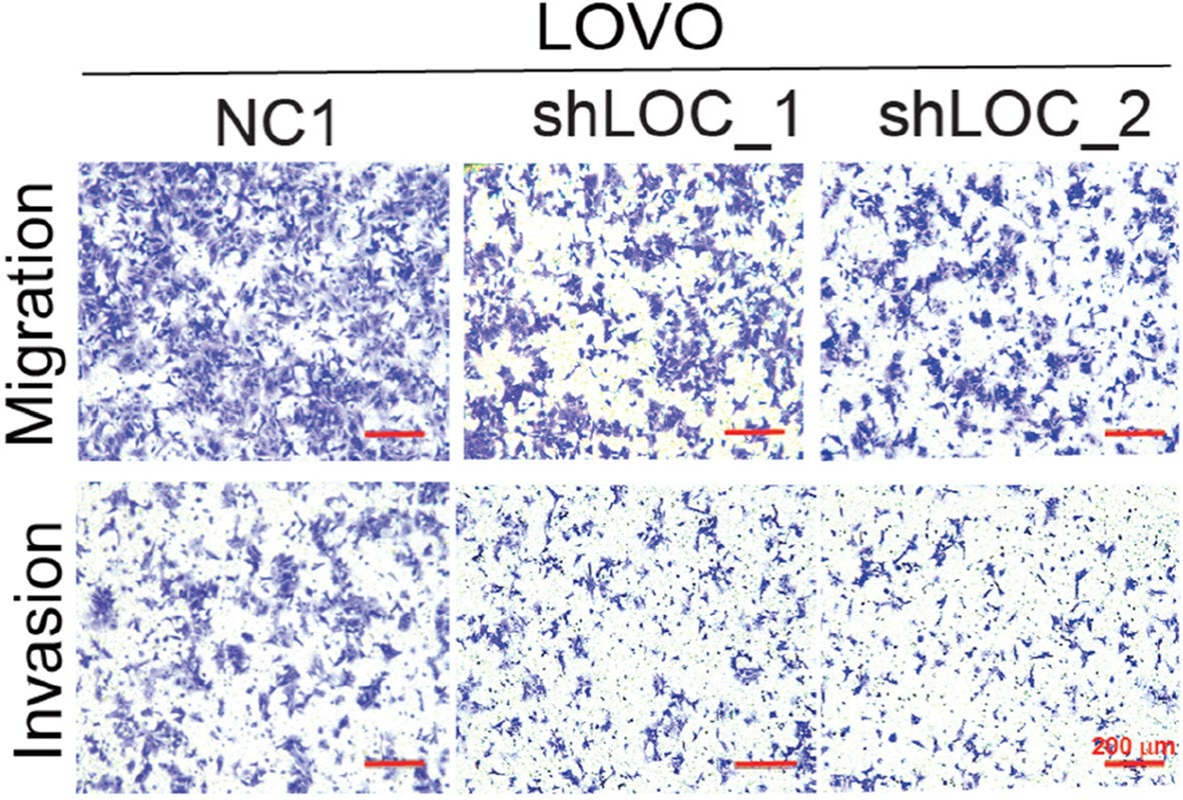
Impact of LOC101927668 on CRC cell proliferation and metastasis. **A**, **B** Assessment of cell proliferative ability through CCK8 in LOC101927668-overexpressing HCT116 cells or LOC101927668-depleted LOVO cells. **C**, **D** Analysis of cell cycle distribution by flow cytometry following LOC101927668 overexpression in HCT116 cells and LOC101927668 knockdown in LOVO cells. **E**, **F** EdU corporation assays were performed in LOC101927668 stably overexpressed or depleted CRC cells. **G**, **H** Colony formation assays were conducted to evaluate the reproductive capacity of CRC cells with overexpressed or silenced LOC101927668. **I**-**L** Evaluation of cell motility via wound healing assays (**I**, **J**) and transwell assays (**K**, **L**) subsequent to LOC101927668 overexpression in HCT116 cells and LOC101927668 depletion in LOVO cells. Data are presented as mean ± SD of at least three independent experiments. **P < 0.01, ***P < 0.001

The corrections do not compromise the validity of the conclusions and the overall content of the article. The original article [1] has been corrected.
