# Supplementary material for: Copy number amplification-induced overexpression of lncRNA LOC101927668 facilitates colorectal cancer progression by recruiting hnRNPD to disrupt RBM47/p53/p21 signaling
Source: J Exp Clin Cancer Res. 2024 Sep 30;43:274. doi: 10.1186/s13046-024-03193-7 (PMC11440719; doi:10.1186/s13046-024-03193-7)
Supplement: Supplementary file 1 — Supplementary Material 1. [file 13046_2024_3193_MOESM1_ESM.docx]

**Supplementary Information**

**Copy number amplification-induced overexpression of lncRNA LOC101927668 facilitates colorectal cancer progression by recruiting hnRNPD to disrupt RBM47/p53/p21 signaling**

Zaozao Wang^1†*^, Haibo Han^2†^, Chenghai Zhang^1†^, Chenxin Wu^1†^, Jiabo Di^1^, Pu Xing^1^, Xiaowen Qiao^1^, Kai Weng^1^, Hao Hao^1^, Xinying Yang^1^, Yifan Hou^1^, Beihai Jiang^1^, Xiangqian Su^1*^

†These authors contributed equally to this work.

*Corresponding authors.

**Table S1. Sequences of shRNA and siRNAs**

| **Name** | **Sequences** |
| --- | --- |
| shLOC101927668-1 | 5’-GTTCCAGAGAGAAATTCACACTGTTCAAGAGACAGT  GTGAATTTCTCTCTGGAACTTTTT-3’ |
| shLOC101927668-2 | 5’-CAAGAGATATGGAACTGAACTTCTTCAAGAGAGAAG  TTCAGTTCCATATCTCTTGTTTTT-3’ |
| shRBM47-1 | 5’-CCGTGCATGCCATGAACAATTCAAGAGATTGTTCATG  GCATGCACGGTTTTT-3’ |
| shRBM47-2 | 5’- TCAACCCAGTTGCCATTAATTCAAGAGATTAATGGCA  ACTGGGTTGATTTTT-3’ |
| sihnRNPD-1 | Sense: 5’-AGACUGCACUCUGAAGUUATT-3’ |
| sihnRNPD-2 | Sense: 5’-GAAGGUGAUUGAUCCUAAATT-3’ |

**Table S2. Sequences of primers**

**Primers used for RT-qPCR and molecular cloning**

| **Name** | **Sequences** |
| --- | --- |
| GAPDH | F: TGCACCACCACCTGCTTAGC |
|  | R: GGCATGGACTGTGGTCATGAG |
| RNU6 | F: TCGCTTCGGCAGCACAT |
|  | R: TGGAACGCTTCACGAATTTGC |
| LOC101927668 | F: GCATTTCCAGTTCCAGAGAG |
|  | R: CACGGGTATGTTTCAGGAC |
| RBM47 | F: TGATGGACTTTGACGGCAAGA |
|  | R: GGGCGGATCTCGTAGTTGTT |
| P53 | F: CAGCACATGACGGAGGTTGT |
|  | R: TCATCCAAATACTCCACACGC |
| P21 | F: CGATGGAACTTCGACTTTGTCA |
|  | R: GCACAAGGGTACAAGACAGTG |
| SESN1 | F: CTACATTGGAATAATGGCTGCGG |
|  | R: AGGTCTATGGGCTAACACTTTGT |
| ZMAT3 | F: CCTTACTTCAATCCCCGCTCT |
|  | R: CTTCGCCAGCTCCAACATTAC |
| TP53I3 | F: GGAGGACCGGAAAACCTCTAC |
|  | R: CCTCAAGTCCCAAAATGTTGCT |
| CD82 | F: GCTCATTCGAGACTACAACAGC |
|  | R: GTGACCTCAGGGCGATTCA |
| HNRNPD | F: GCGTGGGTTCTGCTTTATTACC |
|  | R: TTGCTGATATTGTTCCTTCGACA |
| 7SL1 | F: GCGCGTGCCTGTAGTCCCAGC |
|  | R: ACCTGCTCCGTTTCCGACCTG |
| RNA18S1 | F: GCAAATTACCCACTCCCGACCC |
|  | R: GCCCTCCAATGGATCCTCGTT |
| LOC101927668 full length  (molecular cloning) | F: CCGGAATTCACACCTGACCTCTTCAGTTC |
|  | R: CCGCTCGAGGTTACTTGATTCCCAGTCTTAC |
| LOC101927668-Δ2 | Forward: CCGGAATTCTTAGACTTTGACAAGATGC |
| (molecular cloning) | Reverse: CCGCTCGAGACTCAGTTCTCTCACACAGGT |

**Primers of DNA templates for *in vitro* transcription**

| **Name** | **Sequences** |
| --- | --- |
| LOC101927668 FL | F: TAATACGACTCACTATAACACCTGACCTCTTCAGTTC |
| (1-828 bp) | R: GTTACTTGATTCCCAGTCTTAC |
| LOC101927668-Δ1 | F: TAATACGACTCACTATAACACCTGACCTCTTCAGTTC |
| (1-195 bp) | R: GTTCAACAATGTCTTTATTC |
| LOC101927668-Δ2 | F: TAATACGACTCACTATATTAGACTTTGACAAGATGC |
| (196-373 bp) | R: ACTCAGTTCTCTCACACAGGT |
| LOC101927668-Δ3 | F: TAATACGACTCACTATACCTGAAACATACCCGTGTT |
| (374-639 bp) | R: GTATTTAGCAATCCAGGGATG |
| LOC101927668-Δ4 | F: TAATACGACTCACTATACATCTGCATCCCTGGATTGC |
| (640-828 bp) | R: GTTACTTGATTCCCAGTCTTAC |
| LOC101927668- | F: ATTTAGGTGACACTATAGTTACTTGATTCCCAGTCTTAC |
| Antisense | R: ACACCTGACCTCTTCAGTTC |

**Table S3.** **Details of 9 significantly upregulated SCNA-harboring lncRNAs identified from 9 CRC patients.**

| **lncRNA** | **Genomic location** | **Ever reported ^a^** | **Availability from**  **public database ^b^** |
| --- | --- | --- | --- |
| ZFAS1 | 20q13.13 | **Chen, X., et al. [1]** | Yes |
| SLCO4A1-AS1 | 20q13.33 | **Yu, J., et al. [2]** | Yes |
| SNHG17 | 20q11.23 | **Bian, Z., et al. [3]** | Yes |
| PELATON | 20q13.13 | **Abutalebi, M., et al. [4]** | Yes |
| PVT1 | 8q24.21 | **Shigeyasu, K., et al. [5]** | Yes |
| SNORA71B | 20q11.23 | Wu, G., et al. [6] | Yes |
| LOC101927668 | 7p21.1 | No | Yes |
| AC007405.8 | 2q31.1 | No | Yes |
| XLOC_007214 | 8q24.21 | No | No |

^a^ If the specific lncRNA has been previously reported in CRC, the reference relevant to CRC is provided and shown in bold; otherwise, the first article reporting the lncRNA with cancer is cited.

^b^ Expression data for the specific lncRNA could be accessed from the TCGA or GEO databases.


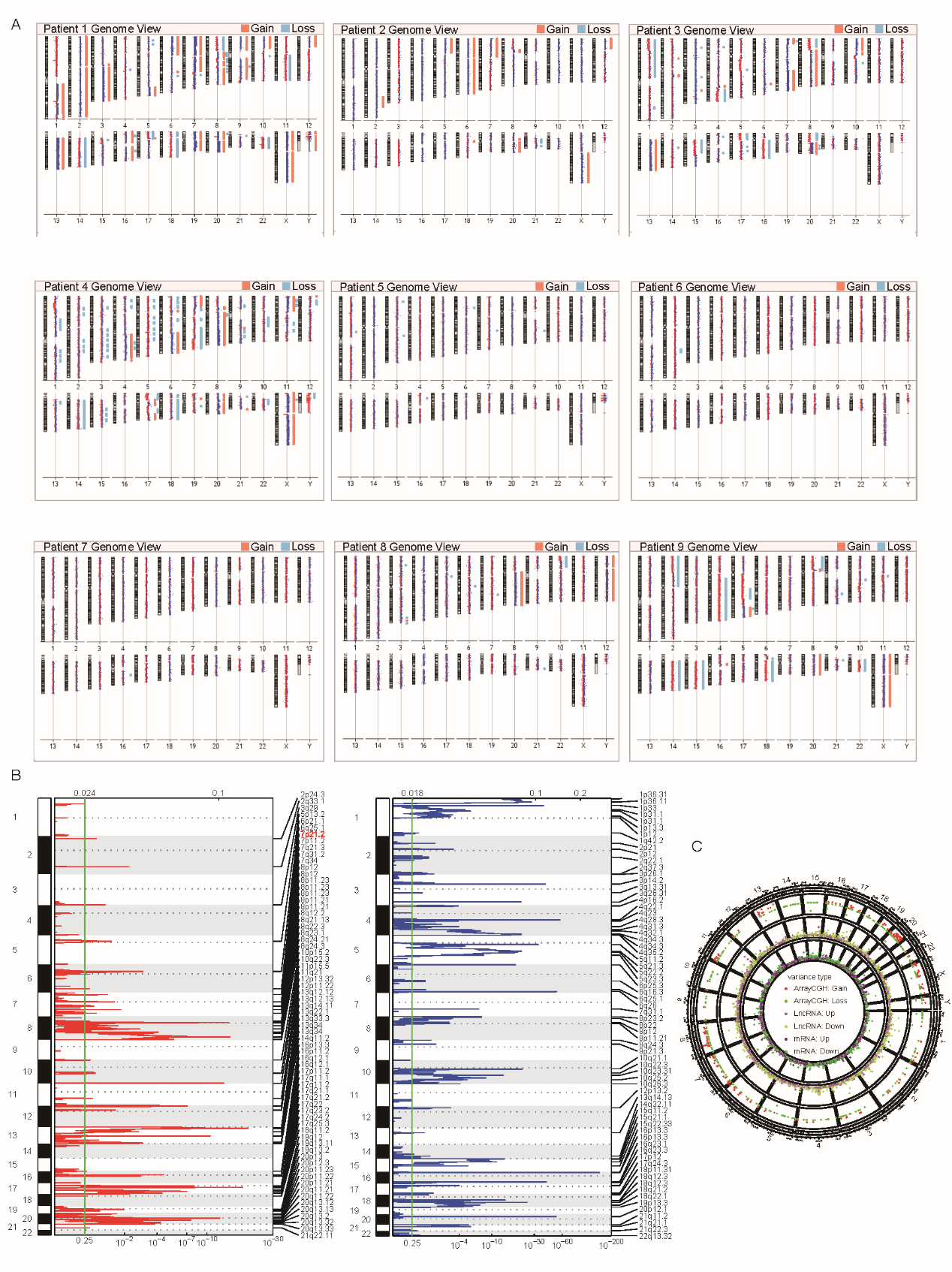


**Figure S1: Characterization of SCNA profiles in whole genomes of CRC patients. A.** Representation of copy number variations in nine CRC patients across chromosomes 1-22 and chromosomes X, Y. Copy number gains are depicted in red, while copy number losses are depicted in blue.

**B.** Overview of genome-wide SCNA patterns in 976 CRC patients sourced from TCGA-COAD database. Regions exhibiting copy number gains are highlighted in red, while regions with copy number losses are highlighted in blue. The green line represents the significance threshold.

**C.** Circos map displaying the integration of SCNA profiles with differentially expressed mRNA and lncRNA profiles from the same nine CRC patients.


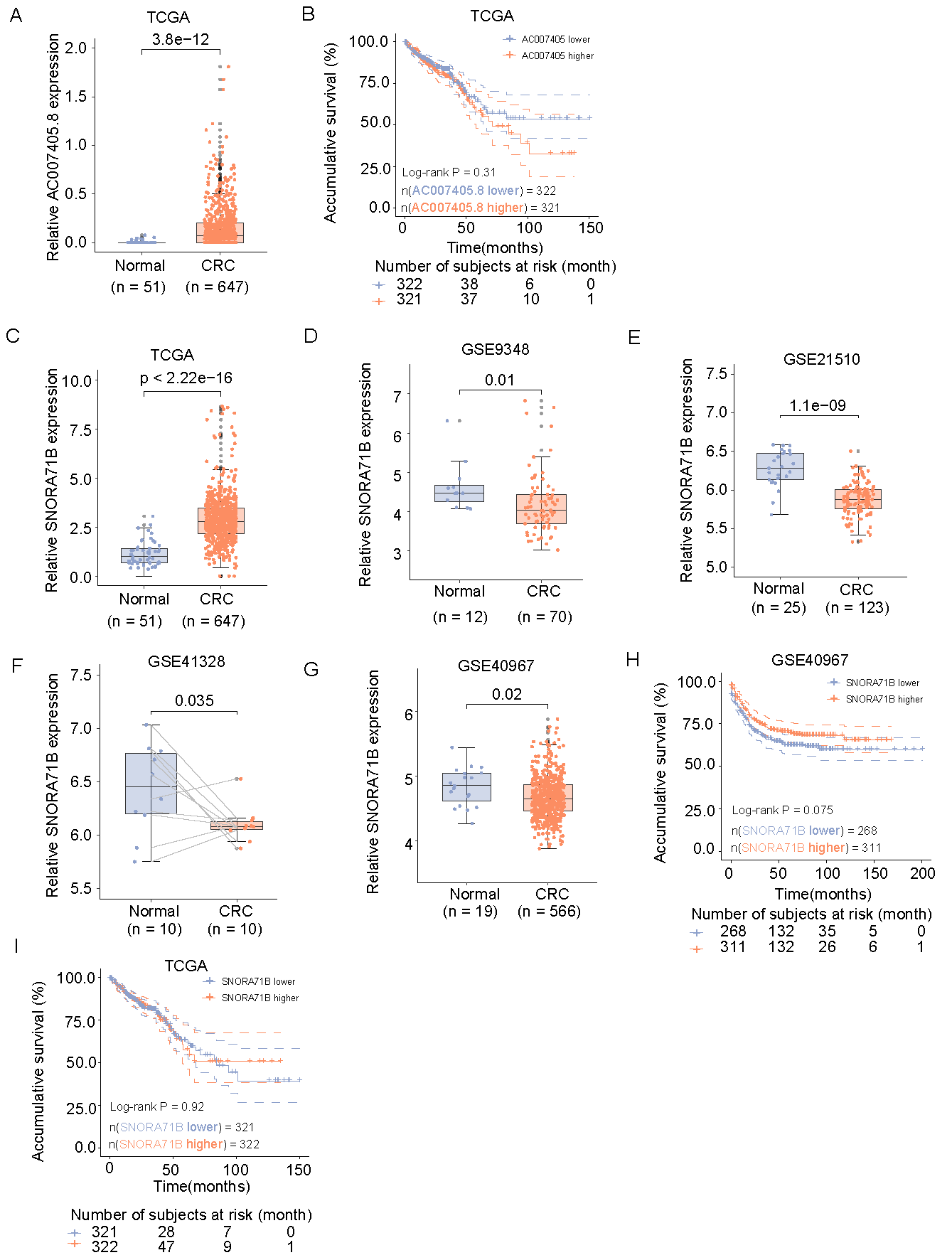


**Figure S2. Expression profiles of AC007405.8 and SNORA71B across TCGA and GEO databases.**

**A.** Expression levels of AC007405.8 in CRC specimens compared to normal tissues in TCGA.

**B.** Kaplan-Meier analysis coupled with log-rank testing was employed to investigate the association between AC007405.8 expression and overall survival among CRC patients in the TCGA dataset.

**C-G.** Expression levels of SNORA71B were evaluated in normal mucosa and CRC tissues in TCGA (C), GSE9348 (D), GSE21510 (E), GSE41328 (F), and GSE40967 (G) datasets.

**H-I.** Kaplan-Meier analysis of overall survival with log-rank test in CRC patients stratified by SNORA71B expression in GSE40967 (H) and TCGA (I) databases.


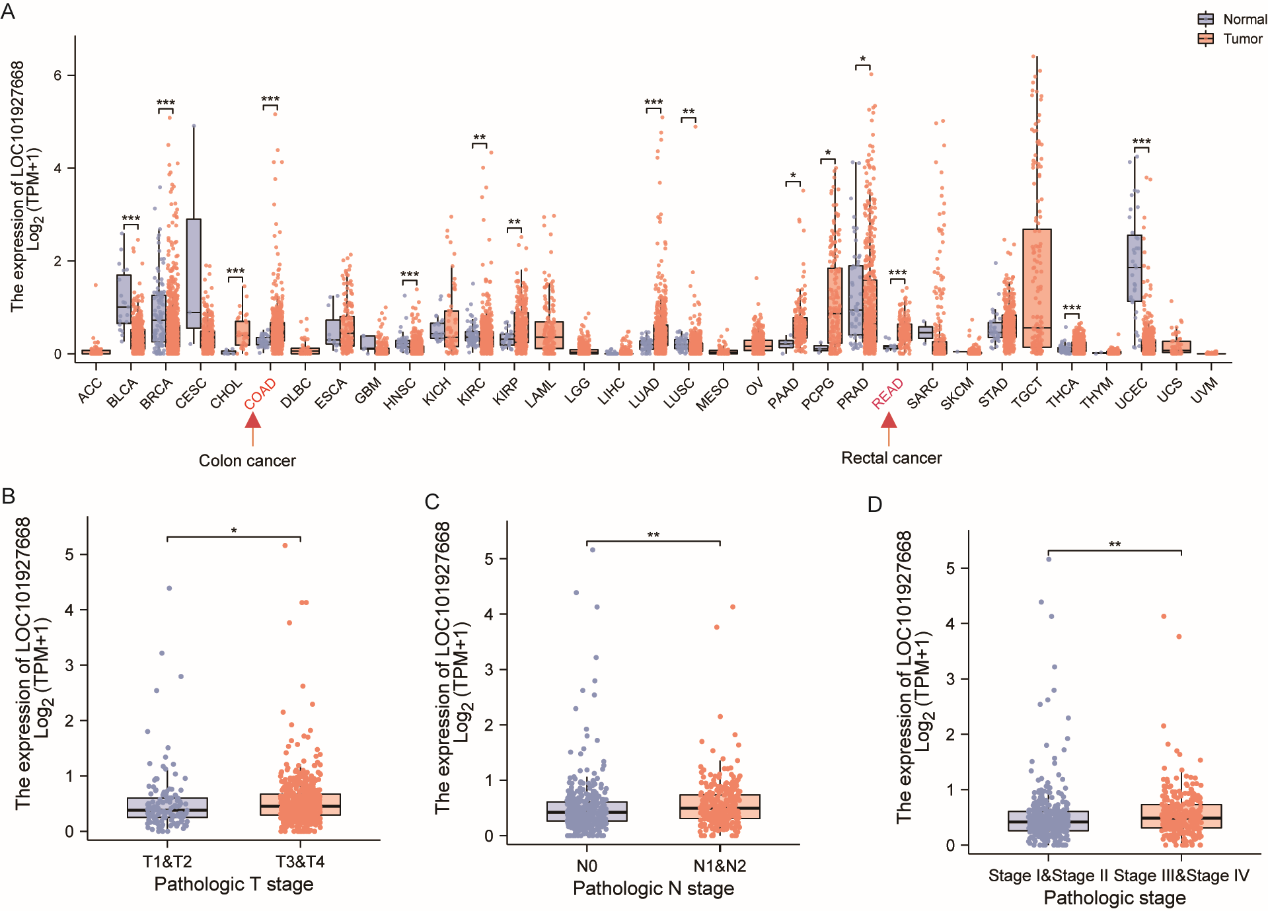


**Figure S3: Upregulation of LOC101927668 in CRC tissues and its association with poor prognosis.**

**A**. Expression levels of LOC101927668 in the pan-cancer dataset from TCGA. Arrows indicated the expression of LOC101927668 in colon cancer (left) and rectal cancer (right) samples compared to their corresponding normal tissues.

**B-D.** Boxplots illustrating the association between LOC101927668 expression levels and different pathologic stages of CRC tumors derived from TCGA data. **P* < 0.05, ***P* < 0.01, ****P <* 0.001.

**
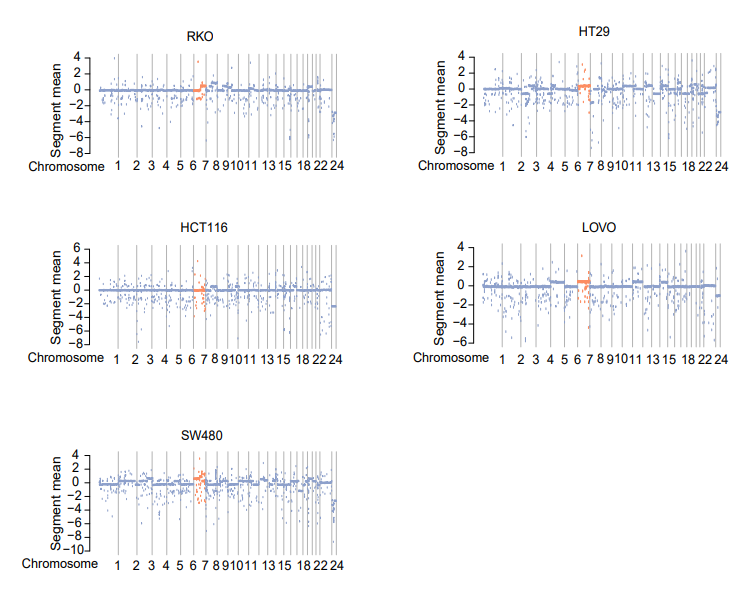
**

**Figure S4. CNV profiles of five CRC cell lines from CCLE.**

Overall copy number variation across the genome of five CRC cell lines (RKO, HT29, HCT116, LOVO, and SW480) downloaded from CCLE database was analyzed by R language via ‘maftools’ package. Chromosome 7 was highlighted with orange.


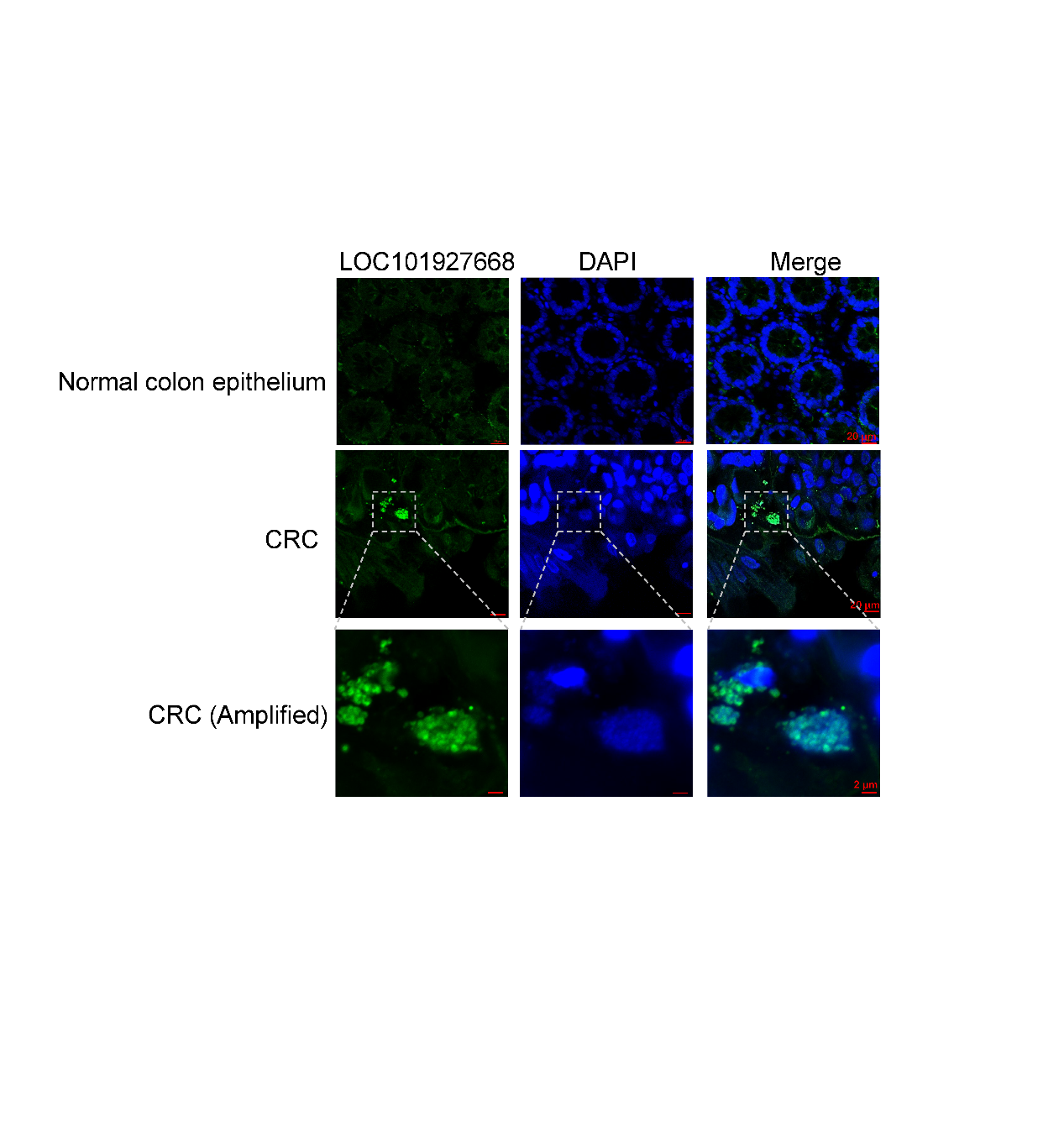


**Figure S5: Subcellular localization of LOC101927668 detected by fluorescence in situ hybridization (FISH) assay in normal colon epithelia and CRC tissues.**

FISH assay was employed to visualize the subcellular localization of LOC101927668 in both normal colon epithelia and CRC tissues. LOC101927668 is represented in green fluorescence, while cell nuclei are stained with DAPI (blue). The upper scale bar represents 20 μm, while the lower scale bar represents 2 μm.


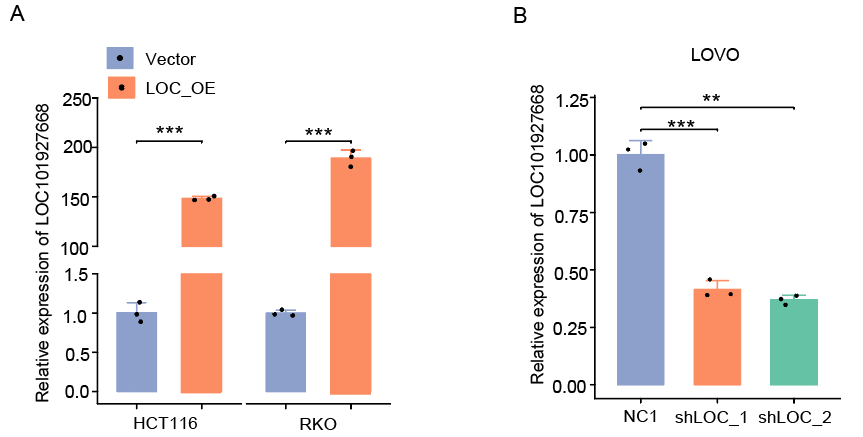


**Figure S6: Assessment of LOC101927668 expression efficiency in LOC101927668-overexpressing HCT116 and RKO Cells, and in LOC101927668-depleted LOVO Cells**.

The efficiency of LOC101927668 expression was quantified using RT-qPCR in HCT116 and RKO cells with stable overexpression (**A**), as well as in LOVO cells with stable depletion (**B**) of LOC101927668. ***P* < 0.01, ****P <* 0.001.

**
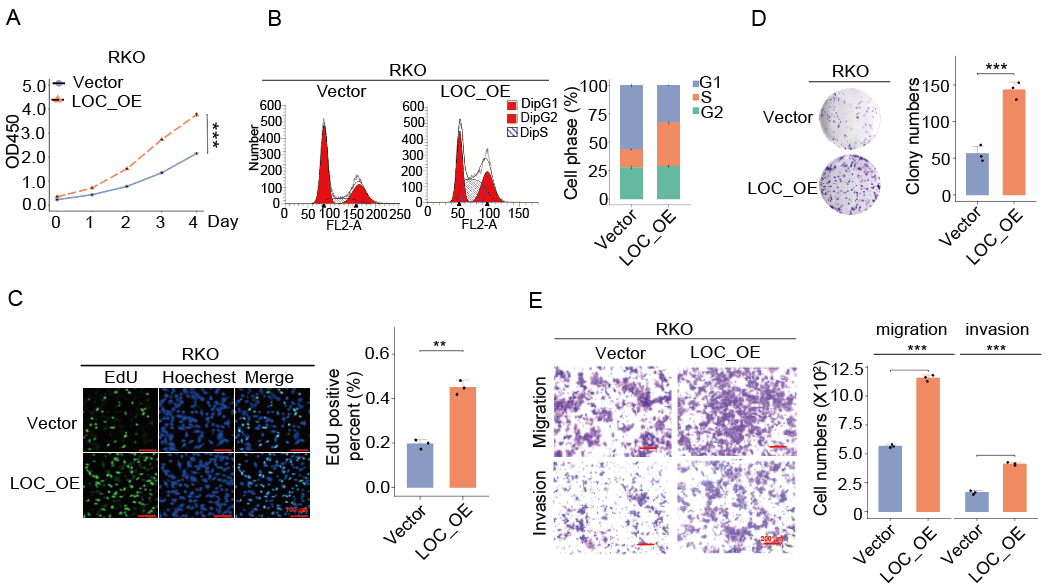
**

**Figure S7: Overexpression of LOC101927668 promoted proliferation and metastasis of RKO cells.**

**A.** Assessment of cell proliferation ability in LOC101927668-overexpressing RKO cells via CCK8 assay.

**B**. Analysis of cell cycle distribution by flow cytometry subsequent to LOC101927668 overexpression in RKO cells.

**C.** EdU incorporation assay was performed in RKO cells with upregulated LOC101927668 or its control vector.

**D.** The reproductive capacity of RKO cells with overexpressed LOC101927668 was evaluated by colony formation assay.

**E**. Evaluation of cell motility using a transwell assay following LOC101927668 overexpression in RKO cells.

Data are presented as mean ± SD of at least three independent experiments. ***P* < 0.01, ****P <* 0.001.


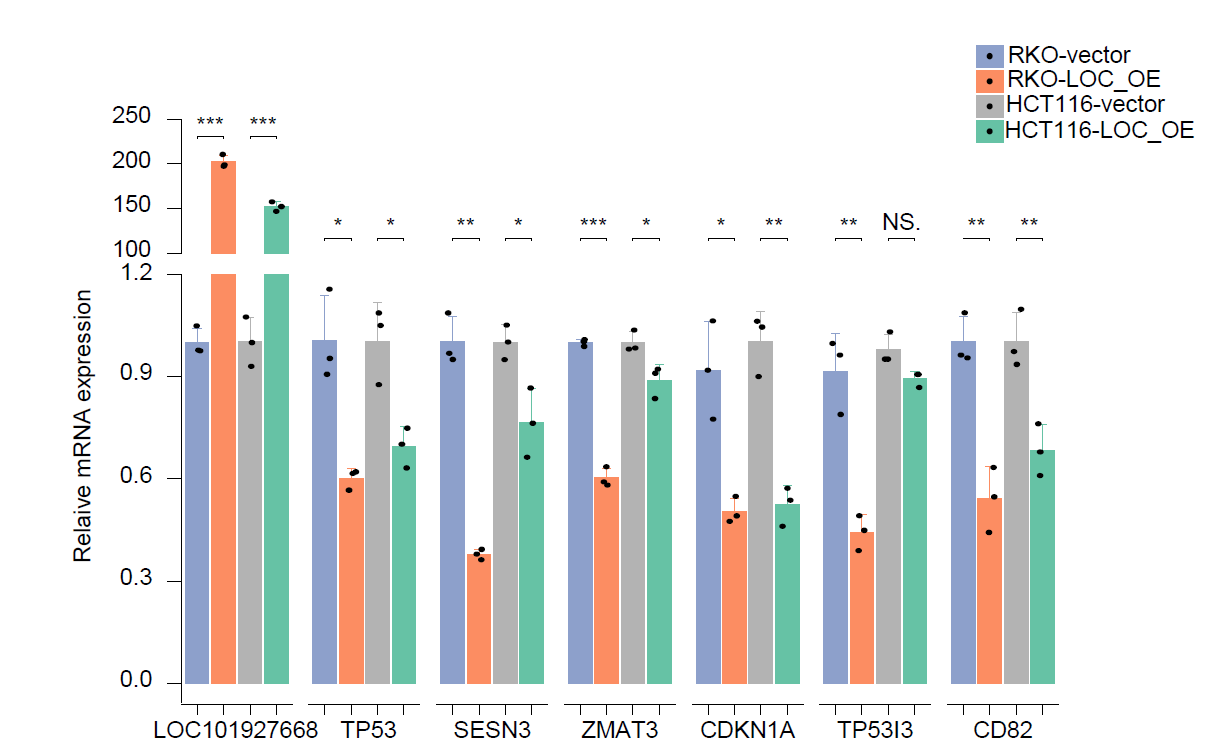


**Figure S8: Evaluation of gene expression changes in the p53 signaling pathway in HCT116 and RKO cells with or without LOC101927668 overexpression.**

The expression levels of genes within the p53 signaling pathway were assessed via RT-qPCR in HCT116 and RKO cells with or without overexpression of LOC101927668. Data are presented as mean ± SD of at least three independent experiments. **P* < 0.05, ***P* < 0.01, ****P* < 0.001.


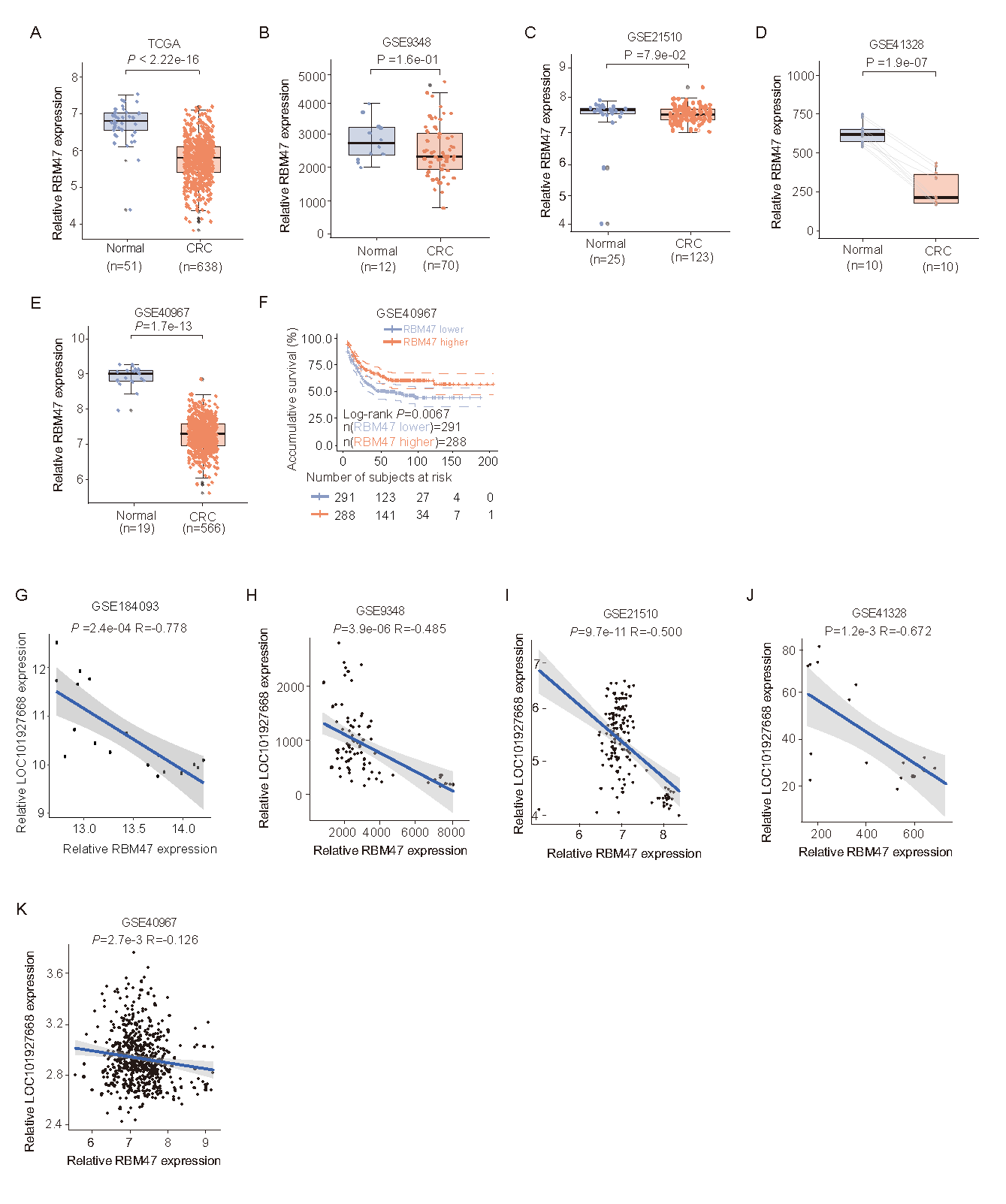


**Figure S9. Expression profiles of RBM47 in CRC samples and its correlation with LOC101927668 across diverse datasets.**

**A-E.** Expression levels of RBM47 were evaluated in normal mucosa and CRC tissues in TCGA (**A**), GSE9348 (**B**), GSE21510 (**C**), GSE41328 (**D**), and GSE40967 (**E**) datasets.

**F**. Kaplan-Meier analysis coupled with log-rank testing was employed to investigate the association between RBM47 expression and overall survival among CRC patients in the GSE40967 dataset.

**G-K.** Pearson correlation analysis was conducted to explore the relationship between LOC101927668 and RBM47 expression in GSE184093 (**G**), GSE9348 (**H**) GSE21510 (**I**), GSE41328 (**J**), and GSE40967 (**K**).


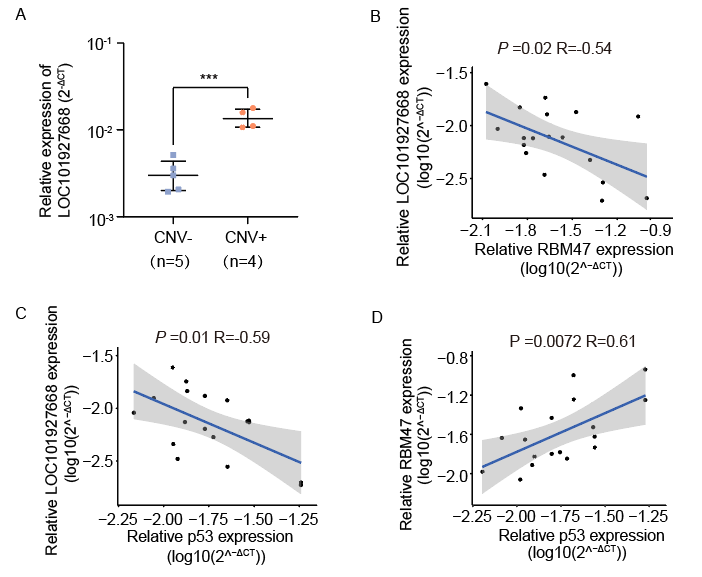


**Figure S10. Analysis of LOC101927668, RBM47, and p53 expression and their correlations in 9 paired CRC tissue samples (GSE184093) using qPCR.**

**A.** LOC101927668 expression was measured in CRC patients with and without chr7p21.1 copy number amplification.

**B-D.** Pearson correlation analysis was conducted to assess the relationship between LOC101927668 and RBM47 expression **(B)**, LOC101927668 and p53 expression **(C)**, and RBM47 and p53 expression **(D)**, respectively.


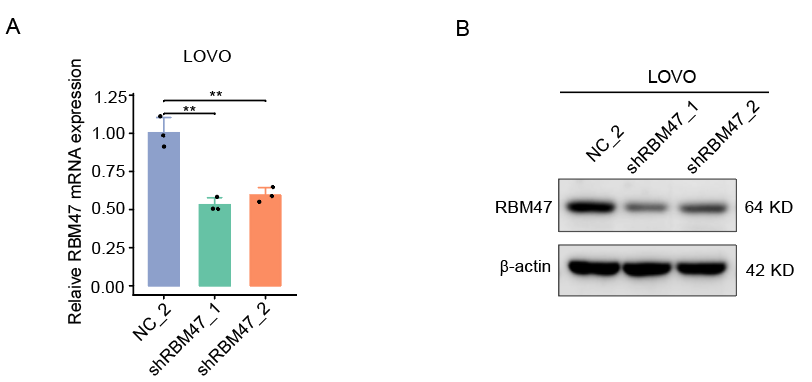


**Figure S11. Assessment of RBM47 shRNA knockdown efficiency in LOVO cells.** The knockdown efficiency of RBM47 shRNA was evaluated using RT-qPCR (**A**) and Western blot analysis (**B**). Data are presented as mean ± SD of at least three independent experiments. ***P* < 0.01, ****P <* 0.001.


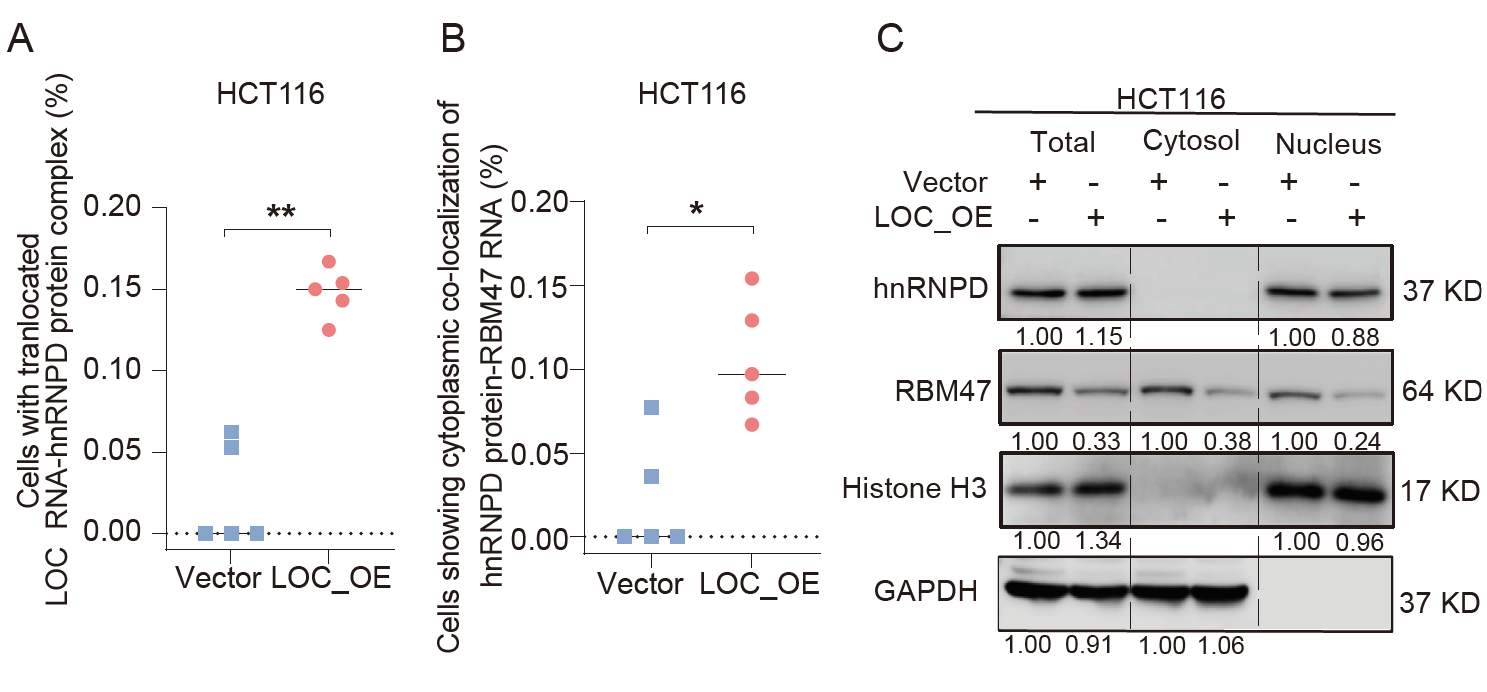


**Figure S12. The proportion of cells containing LOC101927668/RBM47 RNA-hnRNPD protein complexes in HCT116 cells with or without LOC101927668 overexpression, as well as the cytoplasmic and nuclear distribution of hnRNPD and RBM47 protein.**

**A-B.** The proportion of cells exhibiting translocated LOC101927668 RNA-hnRNPD protein complexes in HCT116 cells with or without LOC101927668 overexpression **(A)**, and the proportion of cells showing cytoplasmic co-localization of hnRNPD protein-RBM47 mRNA complexes **(B)** in LOC101927668-overexpressing or control cells, as determined by FISH and IF experiments.

**C.** The nuclear and cytoplasmic protein expression of hnRNPD and RBM47 were analyzed by Western blot. Histone H3 and GAPDH served as internal controls for nuclear and total/cytoplasmic protein, respectively. Quantified densitometry, normalized to their corresponding controls, was presented below the blotting results.


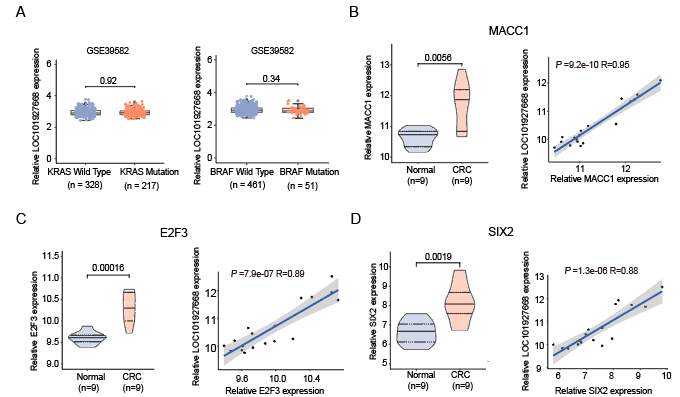


**Figure S13. The impact of KRAS and BRAF mutations on LOC101927668 expression, along with the expression profiles and correlations of certain documented transcription factors with LOC101927668 in CRC.**

1. LOC101927668 expression levels were evaluated in the GSE39582 dataset, grouped by KRAS and BRAF mutation status.

**B-D.** Expression levels and correlations of three reported transcription factors—MACC1 **(B)**, E2F3 **(C)**, and SIX2 **(D)**—with LOC101927668, were analyzed using data from the GSE184093 dataset.

**
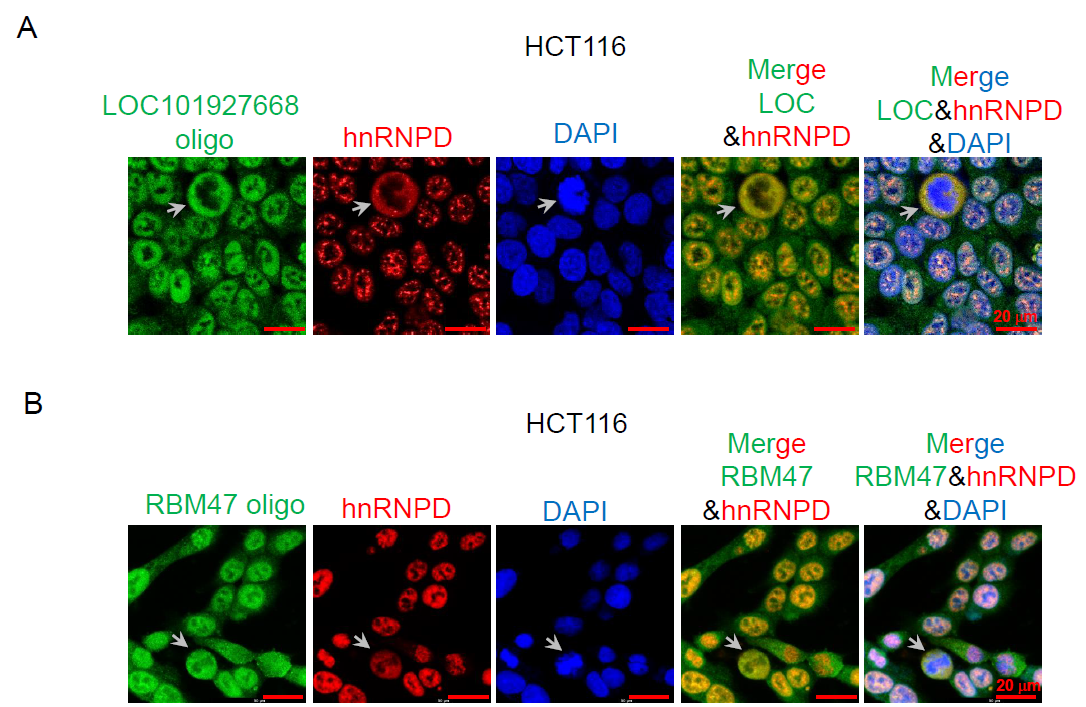
**

**Figure S14. Subcellular localization of LOC101927668 and RBM47 RNA, together with hnRNPD protein was detected by FISH combined with IF in HCT116 cells.**

LOC101927668 ISH combined with hnRNPD IF **(A)**, and RBM47 RNA ISH alongside hnRNPD IF **(B)** were conducted in HCT116 cells. Cells undergoing mitosis were indicated by grey arrows.

**Reference**

1. Chen X, Zeng K, Xu M, Hu X, Liu X, Xu T, et al. SP1-induced lncRNA-ZFAS1 contributes to colorectal cancer progression via the miR-150-5p/VEGFA axis. Cell Death Dis. 2018;9(10):982.

2. Yu J, Han Z, Sun Z, Wang Y, Zheng M, Song C. LncRNA SLCO4A1-AS1 facilitates growth and metastasis of colorectal cancer through beta-catenin-dependent Wnt pathway. J Exp Clin Cancer Res. 2018;37(1):222.

3. Bian Z, Zhou M, Cui K, Yang F, Cao Y, Sun S, et al. SNHG17 promotes colorectal tumorigenesis and metastasis via regulating Trim23-PES1 axis and miR-339-5p-FOSL2-SNHG17 positive feedback loop. J Exp Clin Cancer Res. 2021;40(1):360.

4. Abutalebi M, Li D, Ahmad W, Mokhtari K, Entezari M, Hashemi M, et al. Discovery of PELATON links to the INHBA gene in the TGF-beta pathway in colorectal cancer using a combination of bioinformatics and experimental investigations. Int J Biol Macromol. 2024;270(Pt 1):132239.

5. Shigeyasu K, Toden S, Ozawa T, Matsuyama T, Nagasaka T, Ishikawa T, et al. The PVT1 lncRNA is a novel epigenetic enhancer of MYC, and a promising risk-stratification biomarker in colorectal cancer. Mol Cancer. 2020;19(1):155.

6. Wu G, Hao C, Qi X, Nie J, Zhou W, Huang J, et al. LncRNA SNHG17 aggravated prostate cancer progression through regulating its homolog SNORA71B via a positive feedback loop. Cell Death Dis. 2020;11(5):393.
